# Integrated single-cell and bulk transcriptomic profiling reveals coagulation-associated heterogeneity and prognostic SPINT2 in acute myeloid leukemia

**DOI:** 10.3389/fcell.2026.1971923

**Published:** 2026-10-21

**Authors:** Zongshuai Wang, Min Han, Dejian Zhang, Lisan Cui, Leilei Zhang, Bo Wang

**Affiliations:** Children’s Medical Center, The Second Qilu Hospital of Shandong University, Jinan, China

**Keywords:** acute myeloid leukemia, coagulation related genes, prognosis, risk stratification, SPINT2

## Abstract

**Background:**

Coagulation-associated genes (CARGs) have been implicated in tumor progression and immune regulation, but their cellular heterogeneity and prognostic relevance in acute myeloid leukemia (AML) remain incompletely understood. Here, we sought to characterize the cellular and molecular landscape of CARGs in AML and to identify CARG-associated prognostic determinants with potential functional relevance.

**Methods:**

Single-cell RNA sequencing data were first analyzed to characterize CARG activity across distinct cellular populations in AML. Bulk transcriptomic datasets were subsequently integrated to identify CARG-related molecular subtypes and establish a prognostic scoring system using machine-learning approaches. The associations of the CARG prognostic score with biological pathways, immune characteristics, and drug sensitivity were further evaluated. Single-cell expression patterns, cell–cell communication, and computational perturbation analyses were used to investigate key prognostic genes, followed by experimental validation in AML cells.

**Results:**

Single-cell transcriptomic analysis revealed marked heterogeneity of CARG activity across AML cell populations, with particularly high activity observed in erythroid and cycling erythroid populations. Building on these cellular-level observations, bulk transcriptomic analysis identified three distinct CARG-related molecular subtypes with significantly different survival outcomes and biological characteristics. An integrated machine-learning framework established a seven-gene CARG prognostic score that showed prognostic stratification in both the development and independent external validation cohorts. High CARG prognostic scores were associated with distinct metabolic and immune-related transcriptional programs and differential *ex vivo* responses to several therapeutic agents. Among the model features, *SPINT2* showed prominent prognostic importance and displayed cell-type-specific expression at single-cell resolution. Single-cell analysis revealed cell-type-specific *SPINT2* expression, while computational perturbation analyses suggested associations with erythroid/heme-related and oxidative metabolic programs. Functional experiments further demonstrated that *SPINT2* silencing suppressed AML cell proliferation and promoted apoptosis.

**Conclusion:**

Our study defines a cellular and molecular landscape of CARG heterogeneity in AML and establishes an integrated CARG-based framework for prognostic stratification. The combination of single-cell and bulk transcriptomic analyses, computational perturbation, and functional validation supports *SPINT2* as a candidate gene with functional relevance to AML cell growth and survival and provides a basis for further investigation of coagulation-associated biology in AML.

## Introduction

Acute myeloid leukemia (AML) is a heterogeneous hematologic malignancy characterized by the clonal expansion of immature myeloid progenitor cells in the bone marrow, leading to impaired normal hematopoiesis and rapid disease progression (DiNardo et al., 2023). The pathogenesis of AML involves a complex interplay of genetic and epigenetic alterations, including chromosomal rearrangements and recurrent somatic mutations that disrupt differentiation, proliferation, and survival pathways. Current clinical management relies heavily on risk stratification systems, such as the European LeukemiaNet (ELN) classification, which integrate cytogenetic and molecular abnormalities to guide therapeutic decisions (Duncavage et al., 2022). However, these conventional models exhibit significant limitations, particularly in capturing the biological heterogeneity of AML and predicting individual patient outcomes (Dohner et al., 2022; Park et al., 2024). Patients within the same risk category often display markedly different responses to treatment and survival outcomes, highlighting the need for more refined and biologically informed prognostic models (Dohner et al., 2024). In recent years, increasing attention has been paid to non-genetic determinants, such as microenvironmental signals and systemic biological processes, which may substantially influence disease progression but are not adequately represented in existing models (Sekeres et al., 2026).

The coagulation system is increasingly recognized as a component of the biological landscape of cancer, with coagulation-associated factors participating in inflammation, endothelial interactions, and tumor-associated signaling (Bauer et al., 2022; Mishima et al., 2023; Ahmadi et al., 2023). In AML, dysregulation of coagulation pathways is frequently observed and is clinically manifested by an increased risk of both thrombosis and bleeding complications (Araji et al., 2024; Strasser et al., 2025). Coagulation-related genes (CARGs) are implicated in modulating leukemic cell behavior through multiple mechanisms, including the activation of protease-activated receptors, regulation of endothelial interactions, and promotion of a pro-inflammatory microenvironment (Goyama et al., 2017; McEachron et al., 2010; Nadir et al., 2005; Stucki et al., 2001). Moreover, components of the coagulation cascade have been shown to interact with signaling pathways that govern cell survival and apoptosis, thereby potentially contributing to leukemogenesis and treatment resistance (Sorensen et al., 2003; Langer et al., 2004; Schiller et al., 2002; Paul et al., 2024). Despite these findings, the systematic characterization of CARGs in AML and their integration into predictive models remain insufficiently explored.

The tumor immune microenvironment plays a critical role in the progression and therapeutic response of AML (Chen et al., 2023). The AML bone marrow microenvironment comprises leukemic and non-leukemic cellular populations whose relative abundance, differentiation states, and intercellular interactions contribute to disease heterogeneity (Vadakekolathu and Rutella, 2024; Lion et al., 2012; Spertini et al., 2024; Pawlowska et al., 2001). Furthermore, immune infiltration patterns have been associated with clinical outcomes and responsiveness to emerging immunotherapies, including immune checkpoint inhibitors and cellular therapies (Soleimani Samarkhazan et al., 2025; Valhondo et al., 2025). Recent single-cell transcriptomic studies have revealed substantial cellular and state heterogeneity in AML and have provided a framework for resolving biological programs that may be obscured in bulk transcriptomic analyses (Zhou and Chng, 2024; Soleimani Samarkhazan, 2025). However, the integration of immune infiltration features into robust predictive models for AML remains an ongoing challenge.

Although accumulating evidence highlights the multifaceted roles of CARGs in AML biology, spanning leukemogenesis, immune regulation, and microenvironmental remodeling, their systematic integration into prognostic frameworks and their potential contribution has not been fully characterized. By integrating single-cell and bulk transcriptomic analyses with immune characterization, prognostic modeling, computational perturbation, and functional experiments, we aimed to define the cellular and molecular landscape of CARG-associated heterogeneity in AML, establish a prognostic framework, and identify candidate genes with potential functional relevance.

## Materials and methods

### Data collection and cohort construction

Publicly available transcriptomic profiles and corresponding clinical data for AML were obtained from The Cancer Genome Atlas (TCGA) and Gene Expression Omnibus (GEO) databases. RNA-sequencing data and clinical annotations for the TCGA-LAML cohort were retrieved from the TCGA data portal. Gene identifiers were annotated according to GENCODE v38, and raw read counts were converted to transcripts per million (TPM) values based on gene length and sequencing depth. Duplicated gene identifiers were collapsed to obtain a single gene-level expression value for subsequent analyses. Two additional AML cohorts, GSE37642 and GSE12417, were obtained from the GEO database. GSE37642 and GSE12417 were generated on the GPL96 (Affymetrix Human Genome U133A Array) and GPL570 (Affymetrix Human Genome U133 Plus 2.0 Array) platforms, respectively. Probe identifiers were mapped to official gene symbols using the corresponding platform annotation files. When multiple probes mapped to the same gene, their expression values were averaged to generate a representative gene-level expression value. Each dataset was independently subjected to platform-appropriate preprocessing and normalization before downstream analysis. Patients with available overall survival (OS) time and survival status were included, whereas those lacking essential survival information were excluded. After filtering, 130, 417, and 104 patients with AML were retained in the TCGA-LAML, GSE37642, and GSE12417 cohorts, respectively. TCGA-LAML and GSE37642 were integrated to form the development cohort (n = 547), which was used for molecular subtyping, feature selection, and prognostic model construction, whereas GSE12417 was retained as an independent external validation cohort (n = 104) to evaluate the generalizability of the resulting prognostic model. Because TCGA-LAML and GSE37642 were generated using different transcriptomic platforms, non-biological batch effects were corrected before cohort integration using the ComBat algorithm implemented in the R package sva, with the analysis restricted to genes shared between the two datasets. Principal component analysis (PCA) was performed to assess the global expression structure before and after batch-effect correction. The external validation cohort was independently preprocessed and was not used for batch-parameter estimation, feature selection, parameter tuning, model selection, coefficient estimation, or determination of any model-development parameter.

### Single-cell RNA sequencing data processing and cell-type annotation

Single-cell RNA sequencing (scRNA-seq) data were obtained from the GEO dataset GSE289435. 12 primary AML samples generated using the 10x Genomics platform were included, whereas patient-derived xenograft (PDX) samples were excluded. Raw gene-expression matrices were processed in R using Seurat (v5.5.1), with an independent Seurat object initially created for each patient. Quality control was performed separately for each sample based on the number of detected genes per cell (nFeature_RNA), total unique molecular identifier counts (nCount_RNA), and the proportion of mitochondrial transcripts (percent.mt). Sample-specific filtering thresholds were determined according to the distributions of these quality-control metrics to remove low-quality cells and cells with abnormally high transcriptomic complexity. Potential doublets were identified using DoubletFinder (v2.0.6) and excluded before downstream integration and analysis. Following quality control, gene-expression data were normalized using the LogNormalize method implemented in Seurat, and highly variable genes were identified for dimensionality reduction. The normalized expression matrix was subsequently centered and scaled, followed by PCA. To mitigate technical variation and inter-patient heterogeneity, Harmony (v2.0.5) was applied to the PCA embeddings using patient identity as the batch variable. A shared nearest-neighbor (SNN) graph was then constructed in the Harmony-corrected latent space, followed by unsupervised graph-based clustering. Uniform manifold approximation and projection (UMAP) and t-distributed stochastic neighbor embedding (t-SNE) were used to visualize the cellular composition and cluster structure in reduced-dimensional space. Cell identities were assigned by integrating cluster-specific marker-gene expression with established hematopoietic lineage markers and transcriptional characteristics relevant to AML. Differentially expressed marker genes were first identified for each cluster, after which clusters were manually annotated according to the coordinated expression of canonical lineage markers rather than individual marker genes alone. Patient-level distributions were also examined to exclude annotations driven predominantly by individual samples. Clusters exhibiting closely related transcriptional profiles and consistent lineage-marker expression were subsequently consolidated into major cellular populations. The final annotations were evaluated by comparing UMAP and t-SNE distributions with the expression patterns of representative lineage-specific markers.

### Cell–cell communication analysis

Intercellular communication networks were inferred using CellChat (v2.2.0.9001) with the built-in human ligand–receptor interaction database, CellChatDB.human. CellChat objects were constructed from the quality-controlled and annotated scRNA-seq dataset, with the final cell-type annotations used to define cellular groups for communication inference. To reduce potential bias arising from unequal representation of patients and cell populations, stratified subsampling was performed according to both patient identity and cell type before downstream analysis. Using the normalized gene-expression matrix, overexpressed signaling genes and ligand–receptor interactions were identified within each cellular population, followed by estimation of intercellular communication probabilities. Communication networks were summarized at both the individual ligand–receptor and signaling pathway levels to characterize the number and aggregate strength of interactions among cell populations. Outgoing and incoming signaling strengths were subsequently quantified by integrating pathway-level communication probabilities, thereby estimating the relative contributions of individual cell populations as signal senders and receivers within the inferred communication network. Major signaling pathways were further interrogated to characterize pathway-specific communication patterns, and their contributing ligand–receptor interactions were examined across defined sender–receiver cell pairs. Statistically significant interactions were retained for downstream visualization and identification of prominent intercellular signaling axes.

### CARG-based molecular subtyping and pathway activity analysis

CARGs were curated from coagulation-associated gene sets available in the Molecular Signatures Database (MSigDB), resulting in a predefined set of 39 CARGs for subsequent analyses (Supplementary Table S1). Unsupervised consensus clustering based on the expression profiles of these genes was performed in the AML development cohort using the R package ConsensusClusterPlus. Candidate cluster numbers ranging from k = 2 to 9 were evaluated with 50 resampling iterations. In each iteration, 80% of samples were randomly subsampled while all gene features were retained (pItem = 0.8, pFeature = 1). Partitioning around medoids (PAM) with Euclidean distance was used for clustering, and a fixed random seed was specified to ensure reproducibility. The optimal number of clusters was determined by jointly evaluating the consensus matrices, cumulative distribution function (CDF) curves, and changes in the area under the CDF curve (delta area). The resulting patient groups were designated as CARG molecular subtypes. PCA was performed to visualize the transcriptomic distribution of the identified CARG subtypes. OS across subtypes was estimated using the Kaplan-Meier method, and differences in survival were assessed using the log-rank test. Subtype-specific patterns of CARG expression and clinical characteristics, including survival status, age, and French–American–British (FAB) classification, were visualized using the pheatmap package. Pathway-level differences among the CARG subtypes were further characterized using gene set variation analysis (GSVA). Sample-wise pathway enrichment scores were calculated using the Hallmark gene sets obtained from MSigDB, followed by pairwise comparisons of GSVA scores among the identified subtypes.

### Immune-related profiling and functional enrichment analysis

Immune-related transcriptional features across CARG molecular subtypes and CARG prognostic score-defined risk groups were evaluated using the normalized gene-expression matrix. The ESTIMATE algorithm was applied to calculate the Immune Score, Stromal Score, and ESTIMATE Score for each sample, from which estimated purity was further derived. These metrics were used to characterize relative immune- and stromal-associated transcriptional features across the defined patient groups. Immune cell-associated signatures were further quantified using single-sample gene set enrichment analysis (ssGSEA). Predefined marker gene sets representing 23 immune cell populations were used as reference signatures to calculate sample-specific enrichment scores. These scores were compared across the CARG molecular subtypes and between the high- and low-CARG-score groups, with *P* values adjusted for multiple comparisons using the Benjamini–Hochberg procedure. In parallel, the expression levels of immune regulatory and checkpoint-related genes, including PDCD1, CTLA4, LAG3, and PDCD1LG2, were compared across CARG-defined groups to characterize differences in immune-related regulatory profiles. Differential expression analysis among the CARG molecular subtypes was performed in a pairwise manner using the R package limma. Genes with an absolute log2 fold change (|log2FC|) ≥ 1 and a Benjamini–Hochberg-adjusted *P* value <0.05 were considered differentially expressed genes (DEGs). DEGs identified from each pairwise subtype comparison were subsequently intersected using VennDiagram, and genes shared across the comparisons were retained for downstream functional annotation. Gene Ontology (GO) and Kyoto Encyclopedia of Genes and Genomes (KEGG) enrichment analyses of the shared DEGs were performed using the R package clusterProfiler.

### Development and SHAP interpretation of the CARG prognostic model

The 243 shared DEGs identified across the pairwise comparisons of the CARG molecular subtypes were first subjected to univariable Cox proportional hazards regression analysis in the development cohort. Genes significantly associated with overall survival (*P* < 0.05) were retained, yielding 30 candidate prognostic genes for subsequent model development. A CARG-based prognostic model was then developed by applying multiple survival machine-learning approaches to these candidate genes. Nine classes of survival-learning algorithms were considered, including least absolute shrinkage and selection operator Cox regression (LASSO-Cox), Ridge-Cox, elastic net Cox regression (Enet), stepwise Cox regression (StepCox), CoxBoost, survival support vector machine (survivalSVM), supervised principal components (SuperPC), partial least squares regression for Cox models (plsRcox), and gradient boosting machine (GBM). StepCox was implemented using forward, backward, and bidirectional variable-selection strategies, whereas Enet models were evaluated across multiple α values to vary the relative contributions of L1 and L2 regularization. By incorporating alternative algorithm-specific parameter settings and sequential combinations of complementary modeling approaches, a total of 68 candidate modeling strategies were evaluated. Candidate modeling strategies were ranked according to their C-index performance in the development cohort, which served as the primary criterion for model selection. C-indices obtained in the external validation cohort and the corresponding cross-cohort average C-indices were additionally summarized for descriptive comparison and were not used as the model-selection criterion. All model training, feature selection, parameter tuning, and model selection procedures were performed exclusively within the development cohort. Prognostic discrimination was assessed using the concordance index (C-index). After the model architecture, selected features, and model parameters were finalized, the resulting model was locked and subsequently applied to the independent external validation cohort without further modification. For each patient, a CARG prognostic score was calculated from the linear predictor of the finalized model. Specifically, the CARG prognostic score was calculated as follows: CARG prognostic score = (−0.0074 × AGTPBP1) + (1.0348 × TMEM38B) + (2.0164 × *SPINT2*) + (0.7373 × SH3BP5) + (0.5050 × LAPTM4B) + (0.2137 × HIST1H4H) − (1.0218 × SLC35C2), where each term represents the corresponding gene-expression value multiplied by its model coefficient. Within each cohort, patients were stratified into high- and low-CARG-score groups using the cohort-specific median CARG prognostic score as the cutoff. The scoring formula and model coefficients derived from the development cohort were applied unchanged to the external validation cohort, without additional feature selection, model refitting, or coefficient re-estimation. The cohort-specific median cutoff was used only for dichotomized survival stratification. OS was estimated using the Kaplan-Meier method, and survival differences between the high- and low-score groups were assessed using the log-rank test. Time-dependent receiver operating characteristic (ROC) analysis was performed to evaluate the discriminatory performance of the CARG prognostic score for 3-, 5-, and 7-year OS, with the corresponding areas under the curve (AUCs) calculated. CARG prognostic score distributions were also compared across the identified CARG molecular subtypes to evaluate the relationship between continuous patient-level risk estimates and CARG-based subtype classification. To interpret the contribution of individual model features to prognostic predictions, the finalized model was further evaluated using the SHapley Additive exPlanations (SHAP) framework. The Cox linear predictor generated by the locked model was used as the prediction target, and patient-level SHAP values were calculated for each model gene. Global feature importance was quantified using the mean absolute SHAP value (mean |SHAP value|), which was used to rank genes according to their overall contributions to model predictions. SHAP summary plots were further generated to visualize the relationship between gene-expression levels and both the direction and magnitude of their contributions to predicted risk. Positive SHAP values indicated that a feature shifted the Cox linear predictor toward higher estimated risk, whereas negative SHAP values indicated a shift toward lower estimated risk. SHAP analysis was performed exclusively for *post hoc* interpretation of the finalized prognostic model and was not used for feature selection, model selection, parameter tuning, or risk-cutoff determination.

### Clinical prognostic evaluation and nomogram development

To evaluate the clinical relevance of the CARG prognostic score, patients with available French–American–British (FAB) morphology information were categorized according to FAB M3 status. The M3 group comprised cases annotated as conventional FAB M3 or the microgranular variant M3v, both corresponding to acute promyelocytic leukemia (APL)-associated morphology, whereas M0, M1, M2, M4, M5, M6, and M7 were classified as non-M3 AML. Patients with missing or unclassified FAB information were excluded from FAB-based analyses. M3 was evaluated separately because APL represents a clinically and biologically distinct AML entity compared with other FAB subtypes. CARG prognostic score distributions were then compared between the M3 and non-M3 groups and between age groups (<65 vs. ≥65 years). Cox proportional hazards regression analyses were subsequently performed to assess the associations of FAB M3 status (M3 vs. non-M3), age, and the CARG prognostic score with OS. Univariable Cox regression was first conducted for each variable, followed by multivariable Cox regression incorporating the CARG prognostic score together with the evaluated clinical covariates. Hazard ratios (HRs), 95% confidence intervals (CIs), and *P* values were calculated to quantify their associations with OS. Based on the variables retained in the multivariable model, age and the CARG prognostic score were incorporated into a prognostic nomogram using the R package rms to estimate 3-, 5-, and 7-year OS probabilities. Internal validation of the nomogram was performed using 1,000 bootstrap resamples. Calibration curves were generated to assess agreement between predicted and observed survival probabilities, and calibration performance was further quantified using the Brier score and expected-to-observed (E/O) ratio. Time-dependent concordance index (C-index) analysis was used to evaluate prognostic discrimination over follow-up and to compare the discriminatory performance of age alone with that of the CARG prognostic score. Decision curve analysis (DCA) was further performed to assess the potential incremental clinical utility of incorporating the CARG prognostic score into prognostic assessment. An age-only model and a combined model including both age and the CARG prognostic score were constructed, and net benefit was estimated across a range of threshold probabilities for 3-, 5-, and 7-year OS prediction.

### Beat AML 2.0 ex vivo drug sensitivity analysis

*Ex vivo* drug responses in patient-derived AML samples were evaluated using publicly available data from Beat AML 2.0 waves 1–4. Normalized RNA-expression profiles, probit curve-fitted drug-response data, sample mapping information, clinical annotations, and drug family and molecular target annotations were integrated for analysis. Only AML samples with matched RNA-expression, drug-response, and clinical data were included, and analyses were restricted to quality-controlled single-agent treatments. Drug response was quantified using the area under the probit curve (AUC), with lower AUC values indicating greater *ex vivo* drug sensitivity. For each Beat AML sample, the CARG prognostic score was calculated using the predefined seven-gene prognostic model. Expression values of the model genes were standardized and weighted using the coefficients of the locked model, without re-estimation of the model structure or coefficients in the Beat AML cohort. Spearman rank correlation analysis was performed between drug-specific AUC values and either the CARG prognostic score or *SPINT2* expression, with both predictors treated as continuous variables. Negative correlations therefore indicated that higher CARG prognostic score or *SPINT2* expression were associated with greater *ex vivo* drug sensitivity, whereas positive correlations indicated reduced sensitivity. To account for potential non-independence arising from repeated sampling of individual patients, a complementary patient-level analysis was performed. For patients with multiple samples, the median CARG prognostic score, *SPINT2* expression, and drug-specific AUC were calculated across available samples, and the Spearman correlation analysis was repeated using these patient-level summaries. For categorical comparisons, samples were stratified into high- and low-CARG-score groups according to the median CARG prognostic score, and drug AUC distributions were compared using a two-sided Wilcoxon rank-sum test. Drug family and molecular target annotations provided by Beat AML were used to classify CARG-associated compounds into pharmacological classes. Exploratory class-level enrichment was evaluated using a one-sided Fisher’s exact test, with enrichment ratios calculated relative to the distribution of evaluable compounds across drug classes. For visualization, only classes containing at least two evaluable compounds and at least one CARG-associated compound were displayed.

### Dual-algorithm virtual knockout of *SPINT2* and cross-method perturbation integration

To investigate transcriptional regulatory networks potentially associated with *SPINT2* perturbation at single-cell resolution, computational virtual-knockout analyses were independently performed using scTenifoldKnk (v1.0.3) and GenKI (v0.2.2). Analyses were conducted using the integrated whole-atlas scRNA-seq dataset, with all annotated cells retained without prior restriction to specific cell types or cellular states. Accordingly, the resulting perturbation and enrichment profiles were interpreted as whole-atlas transcriptional associations rather than lineage-specific or cell-autonomous downstream effects of *SPINT2*. Candidate genes were required to be detected in at least 1% of cells and consistently expressed in at least three patients, whereas mitochondrial genes were excluded. Subsequent feature selection was based on gene-expression variability, with *SPINT2* explicitly retained throughout the analysis. For scTenifoldKnk, the raw RNA count matrix was used as input, and 2,500 highly variable genes were retained for gene regulatory network construction. The predicted network consequences of *in silico SPINT2* deletion were characterized by comparing reference and knockout regulatory states using tensor decomposition and manifold alignment. Gene-level perturbation was quantified according to perturbation distances in the aligned manifold space and their corresponding standardized statistics. *P* values were adjusted using the Benjamini–Hochberg procedure, and genes with a false discovery rate (FDR) < 0.05 were considered significantly perturbed. GenKI was applied as an independent virtual-perturbation framework. To reduce imbalance in cellular representation across patients and limit computational burden, up to 1,000 cells were randomly sampled from each patient, and 2,500 highly variable genes were retained for model construction. Perturbation magnitude was quantified using the Kullback–Leibler (KL) divergence between the inferred latent regulatory distributions under the reference and *SPINT2*-knockout states, with higher KL scores indicating greater predicted perturbation. To evaluate the stability of gene rankings, 200 permutation runs were performed and aggregated using a rank-bagging strategy. Genes ranking within the top 5% of perturbed genes in at least 95% of repeated analyses were defined as highly stable perturbation candidates. Cross-method integration was restricted to non-target genes jointly evaluated by both scTenifoldKnk and GenKI. Genes were independently ranked according to scTenifoldKnk perturbation distance and GenKI KL score, and overlap was assessed among the top 100, top 250, and top 500 ranked candidates. Agreement between the two frameworks was further quantified using the Jaccard index for candidate-set overlap and Spearman rank correlation for rank concordance. Consensus perturbation genes were defined as genes ranked within the top 500 by both algorithms or genes reaching FDR <0.05 in scTenifoldKnk while also ranking within the top 500 in GenKI. Using the genes jointly evaluated by both algorithms as the background universe, over-representation analysis (ORA) was subsequently performed for the consensus perturbation genes using MSigDB Hallmark, Kyoto Encyclopedia of Genes and Genomes (KEGG), Reactome, and Gene Ontology Biological Process (GO-BP) gene sets. This cross-method enrichment framework was used to identify biological programs consistently implicated by the two independent computational *SPINT2* perturbation analyses.

### Cell culture

The human AML cell lines THP-1 (ATCC TIB-202), Kasumi-1 (ATCC CRL-2724), HL-60 (ATCC CCL-240), and KG-1 (ATCC CCL-246), together with the human embryonic kidney-derived 293T cell line (ATCC CRL-3216), were obtained from the American Type Culture Collection (ATCC, Manassas, VA, USA). MOLM-13 (DSMZ ACC 554) and OCI-AML3 (DSMZ ACC 582) cells were obtained from the German Collection of Microorganisms and Cell Cultures (DSMZ, Braunschweig, Germany). THP-1 cells were maintained in RPMI-1640 medium (Gibco, Thermo Fisher Scientific, Cat. No. 11875093) supplemented with 10% fetal bovine serum (FBS; Gibco, Thermo Fisher Scientific, Cat. No. 10099141) and 0.05 mM 2-mercaptoethanol (Gibco, Thermo Fisher Scientific, Cat. No. 21985023). MOLM-13 cells were cultured in RPMI-1640 medium supplemented with 10% heat-inactivated FBS, whereas Kasumi-1 cells were maintained in RPMI-1640 medium supplemented with 20% FBS. HL-60 and KG-1 cells were cultured in Iscove’s Modified Dulbecco’s Medium (IMDM; Gibco, Thermo Fisher Scientific, Cat. No. 12440053) supplemented with 20% FBS. OCI-AML3 cells were maintained in Minimum Essential Medium α containing ribonucleosides and deoxyribonucleosides (MEM α, nucleosides; Gibco, Thermo Fisher Scientific, Cat. No. 12571063) supplemented with 20% heat-inactivated FBS. 293T cells were cultured in high-glucose Dulbecco’s Modified Eagle Medium (DMEM; Gibco, Thermo Fisher Scientific, Cat. No. 11965092) supplemented with 10% FBS. When required, FBS was heat-inactivated at 56 °C for 30 min before use. All cells were maintained at 37 °C in a humidified atmosphere containing 5% CO_2_. Suspension AML cell lines were maintained within their recommended density ranges and replenished with fresh medium or passaged every 2–3 days as appropriate, whereas 293T cells were passaged before reaching complete confluence. Only cells in the logarithmic growth phase were used for subsequent experiments. Cell cultures were routinely screened for *mycoplasma* contamination using a PCR-based assay, and only mycoplasma-negative cultures were used.

### Reverse transcription quantitative PCR

*SPINT2* mRNA expression was quantified using reverse transcription quantitative PCR (RT-qPCR). Total RNA was extracted from cells in the logarithmic growth phase using TRIzol Reagent (Invitrogen, Thermo Fisher Scientific, Cat. No. 15596026) according to the manufacturer’s instructions. RNA concentration and purity were assessed before reverse transcription. Complementary DNA (cDNA) was synthesized using HiScript II Q RT SuperMix for qPCR (+gDNA wiper) (Vazyme, Nanjing, China, Cat. No. R223-01). Quantitative PCR was performed using ChamQ Universal SYBR qPCR Master Mix (Vazyme, Cat. No. Q711-02) on a QuantStudio 5 Real-Time PCR System (Applied Biosystems, Thermo Fisher Scientific). GAPDH was used as the internal reference gene. Primer sequences were as follows: *SPINT2*, forward 5′-AGG​CAG​GAT​TCT​GAA​GAC​CA-3′ and reverse 5′-TCC​TCA​GAG​CGG​TAG​CTG​TT-3′; GAPDH, forward 5′-CAA​CGA​CCA​CTT​TGT​CAA​GCT​C-3′ and reverse 5′-GGT​CTA​CAT​GGC​AAC​TGT​GAG​G-3′. Relative *SPINT2* expression was calculated using the 2^−ΔΔCt^ method. All reactions were performed with technical replicates, and at least three independent biological experiments were conducted.

### Western blot analysis

*SPINT2* protein expression was assessed by Western blotting. Total cellular proteins were extracted using RIPA Lysis and Extraction Buffer (Thermo Fisher Scientific, Cat. No. 89900) supplemented with Halt Protease Inhibitor Cocktail (100×; Thermo Fisher Scientific, Cat. No. 78430). Protein concentrations were determined using the Pierce BCA Protein Assay Kit (Thermo Fisher Scientific, Cat. No. 23225). Equal amounts of protein were separated by SDS-PAGE and transferred onto 0.45-μm polyvinylidene fluoride (PVDF) membranes (Thermo Fisher Scientific, Cat. No. 88518). Membranes were blocked with 5% non-fat milk in Tris-buffered saline containing Tween-20 (TBST) for 1 h at room temperature and subsequently incubated overnight at 4 °C with a rabbit recombinant monoclonal antibody against *SPINT2*/HAI-2 (Abcam, Cat. No. ab128926; 1:1,000) and a mouse monoclonal antibody against GAPDH (Abcam, Cat. No. ab8245; 1:5,000). After washing with TBST, membranes were incubated with the appropriate horseradish peroxidase (HRP)-conjugated secondary antibodies, including goat anti-rabbit IgG H&L (HRP) (Abcam, Cat. No. ab97051) and goat anti-mouse IgG H&L (HRP) (Abcam, Cat. No. ab6789). Protein bands were visualized using SuperSignal West Pico PLUS Chemiluminescent Substrate (Thermo Fisher Scientific, Cat. No. 34580) and captured using a ChemiDoc MP Imaging System (Bio-Rad Laboratories, Hercules, CA, USA). Relative *SPINT2* protein abundance was normalized to GAPDH.

### Lentiviral shRNA-mediated *SPINT2* knockdown

Stable *SPINT2*-knockdown AML cells were generated using a pLKO.1-based lentiviral short hairpin RNA (shRNA) system. A non-targeting shRNA construct served as the negative control (shNC), whereas recombinant pLKO.1 vectors carrying *SPINT2*-targeting shRNAs were used for gene silencing. The *SPINT2*-targeting sequences were synthesized by Merck and were as follows: sh*SPINT2*#1, 5′-CTC​CTG​CAA​TAA​CTT​CAT​CTA-3′; and sh*SPINT2*#2, 5′-CTC​CAG​CGA​TAT​GTT​CAA​CTA-3′. For lentiviral production, 293T cells were co-transfected with the pLKO.1-sh*SPINT2* or corresponding control construct together with the second-generation packaging plasmid psPAX2 (Addgene plasmid #12260) and the VSV-G envelope plasmid pMD2.G (Addgene plasmid #12259). Transfection was performed using Lipofectamine 3000 Transfection Reagent (Invitrogen, Thermo Fisher Scientific, Cat. No. L3000015) according to the manufacturer’s instructions. Lentivirus-containing supernatants were collected after transfection, cleared of cellular debris, and used to transduce target AML cells. Following transduction, cells were selected with puromycin dihydrochloride (Gibco, Thermo Fisher Scientific, Cat. No. A1113803). The appropriate puromycin concentration for each cell line was determined using a preliminary kill-curve assay. Stable sh*SPINT2* and shNC cell populations were subsequently maintained under puromycin selection. *SPINT2* knockdown efficiency was verified by RT-qPCR and Western blotting before downstream functional assays.

### CCK-8 cell proliferation assay

Cell proliferation was evaluated using the CCK-8 Cell Counting Kit (Vazyme, Cat. No. A311-01). Cells in the logarithmic growth phase were seeded into 96-well plates at a density of 3,000–5,000 cells per well, with at least three replicate wells for each experimental condition. Cell proliferation was assessed on days 1, 2, 3, 4, and 5 after seeding. At each indicated time point, 10 μL of CCK-8 reagent was added to each well, followed by incubation at 37 °C for 4 h. Absorbance at 450 nm was measured using a microplate reader. Cell-free wells containing culture medium and CCK-8 reagent were used as blanks, and background absorbance was subtracted from the corresponding experimental values. All experiments were independently repeated at least three times. Because separate wells were used at each measurement time point, time-course CCK-8 data were analyzed using ordinary two-way ANOVA with treatment and time as independent factors, including assessment of the treatment × time interaction. Dunnett’s multiple-comparison test was subsequently used to compare each *SPINT2*-knockdown group with the corresponding shNC group at individual time points.

### EdU incorporation assay

DNA synthesis and cell proliferation were assessed using the BeyoClick Plus EdU-488 Cell Proliferation Kit (Beyotime Biotechnology, Shanghai, China, Cat. No. C0072S). Cells were pulse-labeled with 2 μM 5-ethynyl-2′-deoxyuridine (EdU) for 2 h under the indicated experimental conditions. Following EdU incorporation, cells were fixed with 4% paraformaldehyde for 15–20 min and permeabilized with 0.3% Triton X-100 for 10–15 min. The click-labeling reaction was subsequently performed according to the manufacturer’s instructions. EdU incorporation was quantified by flow cytometry using a CytoFLEX Flow Cytometer (Beckman Coulter Life Sciences, Brea, CA, USA), and data were acquired and analyzed using CytExpert software. The proportion of EdU-positive cells within the gated single-cell population was used to quantify DNA synthesis and proliferative activity. All experiments were independently repeated at least three times.

### Flow cytometric analysis of apoptosis

Apoptosis was evaluated using an Annexin V-FITC/PI Apoptosis Detection Kit (BestBio, Shanghai, China, Cat. No. BB-4101). Following the indicated treatments, cells were harvested, washed twice with phosphate-buffered saline (PBS), and resuspended in 300 μL of 1× binding buffer. Cells were then stained with Annexin V-FITC and propidium iodide (PI) according to the manufacturer’s instructions. Samples were incubated for 15–20 min at room temperature in the dark and analyzed within 1 h using a CytoFLEX Flow Cytometer (Beckman Coulter Life Sciences). Flow cytometric data were analyzed using CytExpert software. Annexin V-positive/PI-negative cells were classified as early apoptotic cells, whereas Annexin V-positive/PI-positive cells were classified as late apoptotic or secondary necrotic cells. Total apoptosis was calculated as the sum of the early- and late-apoptotic populations. All experiments were independently repeated at least three times.

### Statistical analysis

All statistical analyses were performed using R software (v4.5.1) and GraphPad Prism (v10.0). Statistical tests for continuous variables were selected according to data distribution and homogeneity of variance. For comparisons between two groups, a two-sided unpaired Student’s t-test was used for normally distributed data with homogeneous variances, whereas the Wilcoxon rank-sum test was applied when parametric assumptions were not met. For comparisons among more than two groups, one-way analysis of variance (ANOVA) was used when parametric assumptions were satisfied, whereas the Kruskal–Wallis test was used for non-parametric data. For the time-course CCK-8 assays, ordinary two-way ANOVA was performed separately for THP-1 and MOLM-13 cells, with treatment and time included as independent factors and the treatment × time interaction evaluated. Dunnett’s multiple-comparison test was used for comparisons of each sh*SPINT2* group with the corresponding shNC group at individual time points. For analyses involving multiple comparisons across genes, cell types, pathways, or drugs, *P* values were adjusted using the Benjamini–Hochberg procedure, with a false discovery rate (FDR) < 0.05 considered statistically significant. All *in vitro* experiments were independently repeated at least three times, and experimental data are presented as the mean ± SD.

## Results

### Single-cell transcriptomic profiling reveals cell type-specific CARG activity in AML

Single-cell transcriptomic analysis was performed using GEO dataset GSE289435, comprising 12 primary AML samples, to characterize the cellular distribution and activity of CARGs in AML. Following quality control, dimensionality reduction, clustering, and cell-type annotation, seven major cellular populations were identified: myeloid progenitors, monocytes, cycling erythroid cells, erythroid cells, B cells, T cells, and dendritic cells (DCs). These populations exhibited distinct clustering patterns in both t-SNE and UMAP embeddings (Figures 1A,B). Cell-type annotations were further supported by the expression of canonical lineage markers. Monocytes preferentially expressed myeloid-associated genes, including LYZ, FCGR3A, CTSS, and CTSD; T and B cells were characterized by CD3D/CD3E and CD79A/MS4A1, respectively; whereas erythroid and cycling erythroid populations showed prominent expression of erythroid- and proliferation-associated markers, including HBB, HBA1, GATA1, and MKI67 (Figure 1C). Marked variation in cellular composition was observed across AML samples. Myeloid progenitors and monocytes represented the predominant populations in most patients, whereas the relative abundance of erythroid cells, lymphocytes, and DCs varied substantially among individuals (Figure 1D), highlighting pronounced interpatient heterogeneity in the cellular landscape of AML. We next quantified CARG-related transcriptional activity at single-cell resolution using UCell. The CARG UCell activity score varied markedly across cellular populations, with cycling erythroid and erythroid cells displaying the highest relative CARG activity. Heterogeneous CARG activity was also evident among myeloid progenitors, monocytes, and subsets of immune cells (Figure 1E). Projection of CARG UCell activity onto the UMAP embedding further demonstrated that high CARG activity was spatially concentrated within discrete cellular regions rather than uniformly distributed throughout the cellular landscape (Figure 1F). Together, these findings indicate that CARG-related transcriptional activity is highly heterogeneous in AML and preferentially enriched in specific cellular populations and states.

**FIGURE 1:**
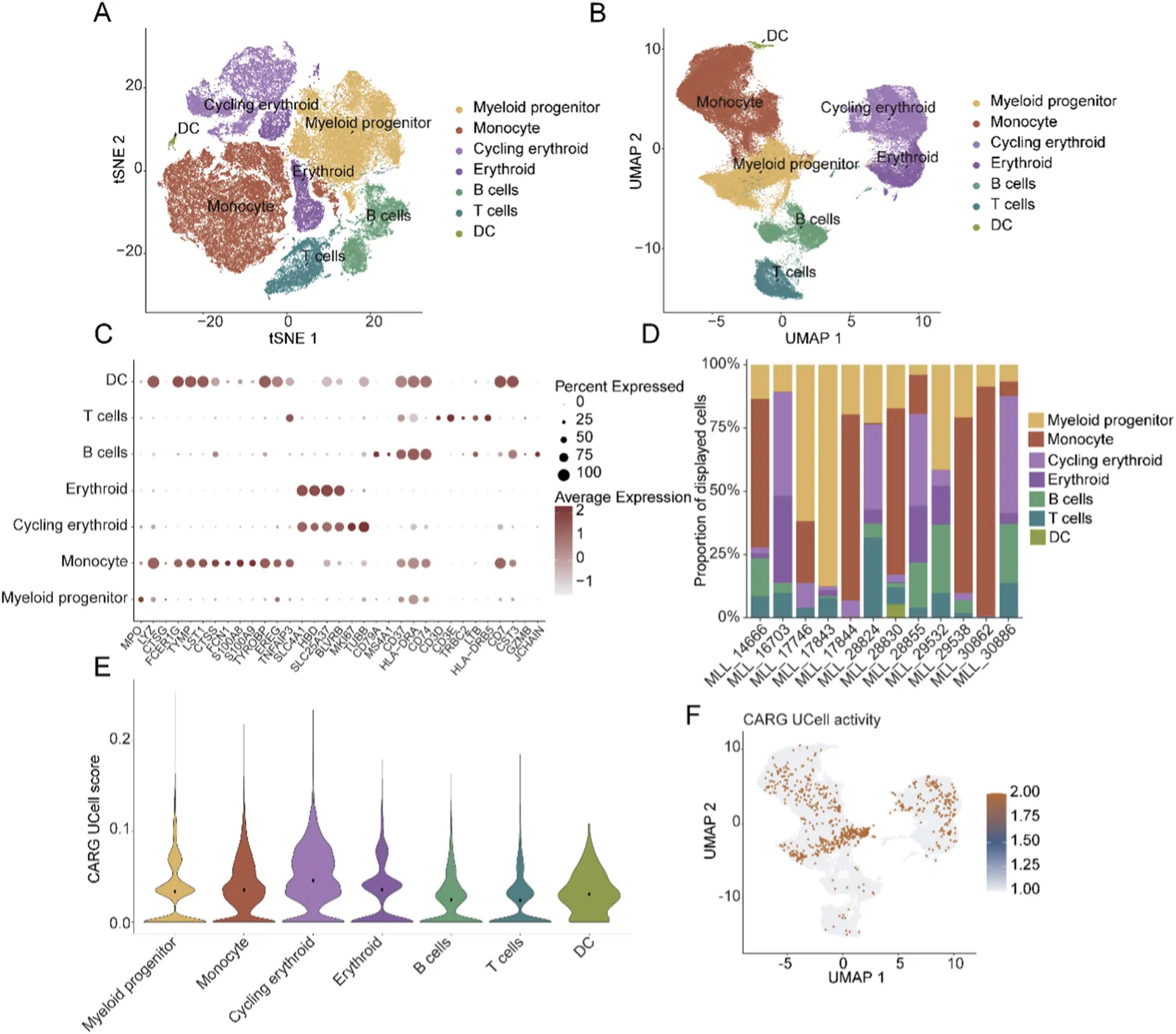
Single-cell transcriptomic analysis reveals cell type-specific distribution of CARG activity in AML. Analyses were based on GEO dataset GSE289435 comprising 12 primary AML samples. **(A, B)** t-SNE and UMAP plots showing the distribution of the major cell populations identified in the AML single-cell transcriptomic dataset. **(C)** Dot plot showing the expression patterns of representative cell-type marker genes across the annotated cell populations. Dot size represents the proportion of cells expressing each gene, and color indicates the average expression level. **(D)** Stacked bar plot showing the relative proportions of different cell types across individual AML samples. **(E)** Violin plots showing the distribution of CARG UCell activity scores across the annotated cell populations. **(F)** UMAP feature plot showing the spatial distribution of CARG UCell activity scores across the single-cell landscape.

### CARG-based molecular subtyping identifies three AML subgroups with distinct prognoses

Bulk transcriptomic analyses were performed in the integrated development cohort comprising TCGA-LAML (n = 130) and GSE37642 (n = 417; total n = 547). Given the heterogeneous CARG-related transcriptional activity observed at the single-cell level, we examined whether CARG expression patterns could define distinct molecular subgroups of AML. Consensus clustering based on CARG expression profiles identified three molecular subtypes: Cluster A (n = 195), Cluster B (n = 217), and Cluster C (n = 135) (Figure 2A). Overall survival differed significantly among the three subtypes (log-rank *P* < 0.001), with Cluster A exhibiting the most favorable prognosis, Cluster C the poorest prognosis, and Cluster B an intermediate outcome (Figure 2B). Principal component analysis further demonstrated distinct transcriptomic distributions among the three subtypes, with Cluster C showing more pronounced separation from Clusters A and B (Figure 2C). Consistent with these findings, the CARG expression heatmap revealed distinct subtype-specific expression patterns, accompanied by differences in survival status, age, and FAB classification across the three subtypes (Figure 2D). These results indicate that CARG-based molecular stratification captures clinically and transcriptionally distinct AML subgroups. To further characterize the biological differences among the CARG subtypes, Hallmark pathway activity was evaluated using GSVA. The three subtypes exhibited broadly distinct pathway-enrichment profiles (Figure 2E). Compared with Cluster B, Cluster A showed higher enrichment of multiple metabolic and cell-state–related programs, including glycolysis, fatty acid metabolism, peroxisome, oxidative phosphorylation, PI3K–AKT–mTOR and mTORC1 signaling, reactive oxygen species pathways, apoptosis, and MYC target programs. By contrast, Cluster B was relatively enriched in heme metabolism, Hedgehog signaling, and selected differentiation-associated pathways. Cluster C also exhibited a distinct functional profile involving pathways associated with heme and lipid metabolism, cell proliferation, cellular stress responses, and immune regulation. Collectively, these findings demonstrate that CARG-based molecular subtyping delineates AML populations with distinct prognostic and pathway-level characteristics, underscoring substantial heterogeneity in CARG-associated biological programs.

**FIGURE 2:**
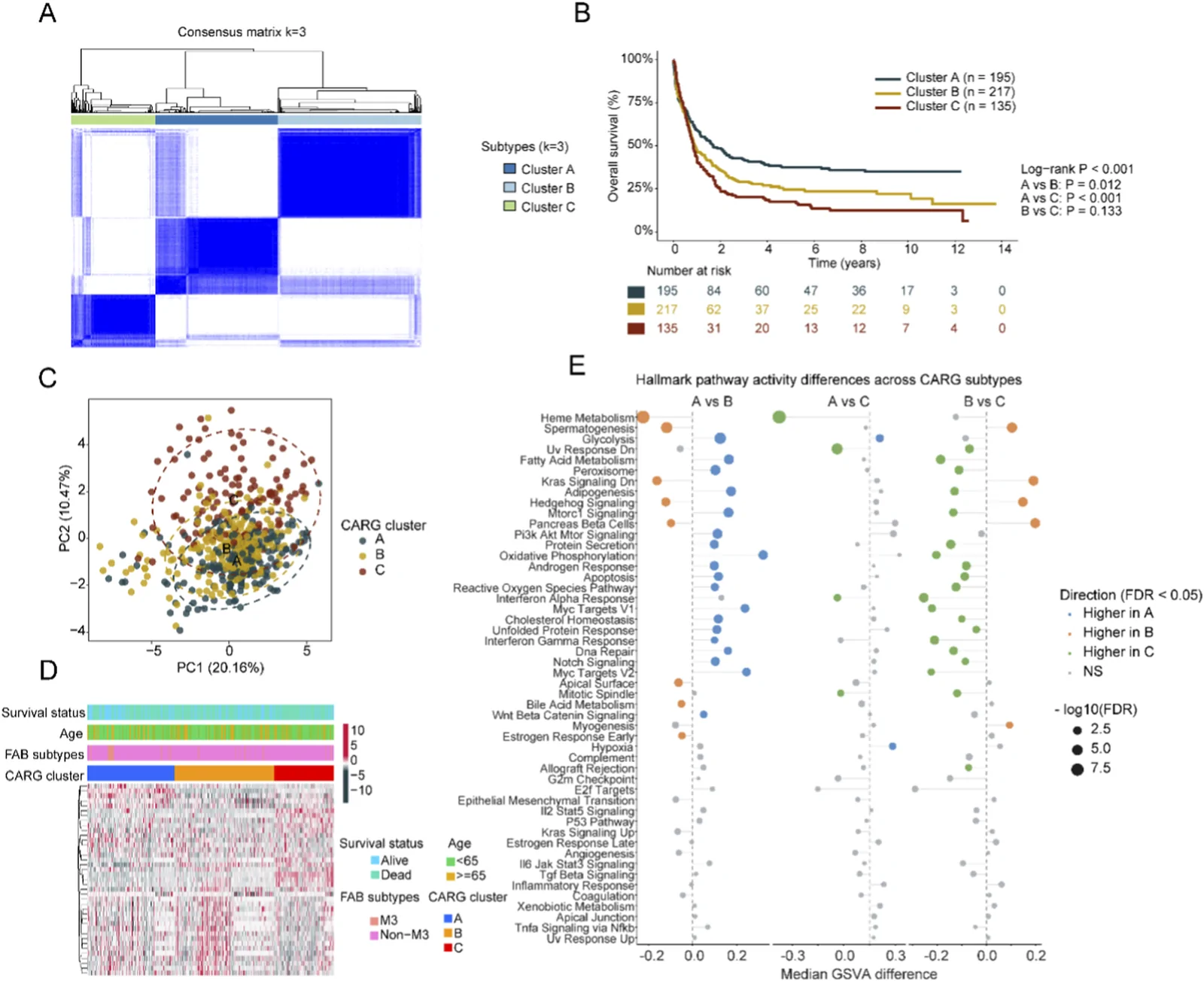
CARG expression patterns define AML molecular subtypes with distinct survival outcomes and pathway activity profiles. Analyses were performed in the integrated development cohort comprising TCGA-LAML (n = 130) and GSE37642 (n = 417; total n = 547). **(A)** Consensus matrix derived from consensus clustering based on CARG expression profiles, with the optimal number of clusters determined as k = 3. **(B)** Kaplan–Meier curves comparing overall survival among the three CARG molecular subtypes. **(C)** Principal component analysis showing the distribution of the three CARG subtypes in transcriptomic space. **(D)** Heatmap showing CARG expression patterns with annotations of clinical characteristics. **(E)** Hallmark gene set-based GSVA comparing pathway activity across CARG subtypes. The x-axis represents the difference in median GSVA scores between the indicated subtype pairs. Colors denote pathways significantly enriched in the corresponding subtype, whereas gray indicates non-significant differences (NS). Dot size represents −log10(FDR), and FDR <0.05 was considered statistically significant.

### CARG-defined AML subtypes exhibit distinct immune-related and coagulation-associated features

Using the same integrated development cohort comprising TCGA-LAML (n = 130) and GSE37642 (n = 417; total n = 547), we further characterized immune-related and functional differences among the CARG-defined molecular subtypes. Cluster B exhibited significantly lower Immune Scores and Stromal Scores than Clusters A and C, accompanied by a lower ESTIMATE Score and higher estimated purity (Figures 3A–D), suggesting a comparatively immune- and stroma-low transcriptional profile. Consistent with these observations, ssGSEA revealed broad differences in immune cell–associated signatures across the three subtypes. Signatures corresponding to activated CD4^+^ T cells, activated CD8^+^ T cells, natural killer cells, regulatory T cells, and several other immune cell populations differed significantly among the groups (Figure 3E). In parallel, multiple immune regulatory genes, including PDCD1LG2, CTLA4, CD40LG, CD80, CD40, CD27, CD28, ICOS, and VTCN1, exhibited distinct subtype-associated expression patterns (Figure 3F), further supporting heterogeneity in immune-related transcriptional states across the CARG subtypes. We next investigated the transcriptional differences underlying these molecular subtypes. Differential expression analysis identified 243 genes shared across the pairwise subtype comparisons (Figure 3G). Gene Ontology enrichment analysis showed that these shared differentially expressed genes were prominently enriched in biological processes related to blood coagulation, coagulation, and hemostasis, together with cellular components and molecular functions involving platelet α-granules, fibrillar collagen, and regulation of proteolytic activity (Figure 3H). KEGG enrichment analysis further highlighted platelet activation, NF-κB signaling, and the pentose phosphate pathway, with platelet activation representing one of the more prominent enriched pathways (Figure 3I). Together, these findings indicate that CARG-defined AML subtypes are distinguished by both immune-related transcriptional features and coagulation-, hemostasis-, and platelet-associated molecular programs, further linking CARG-based stratification to biologically distinct states of AML.

**FIGURE 3:**
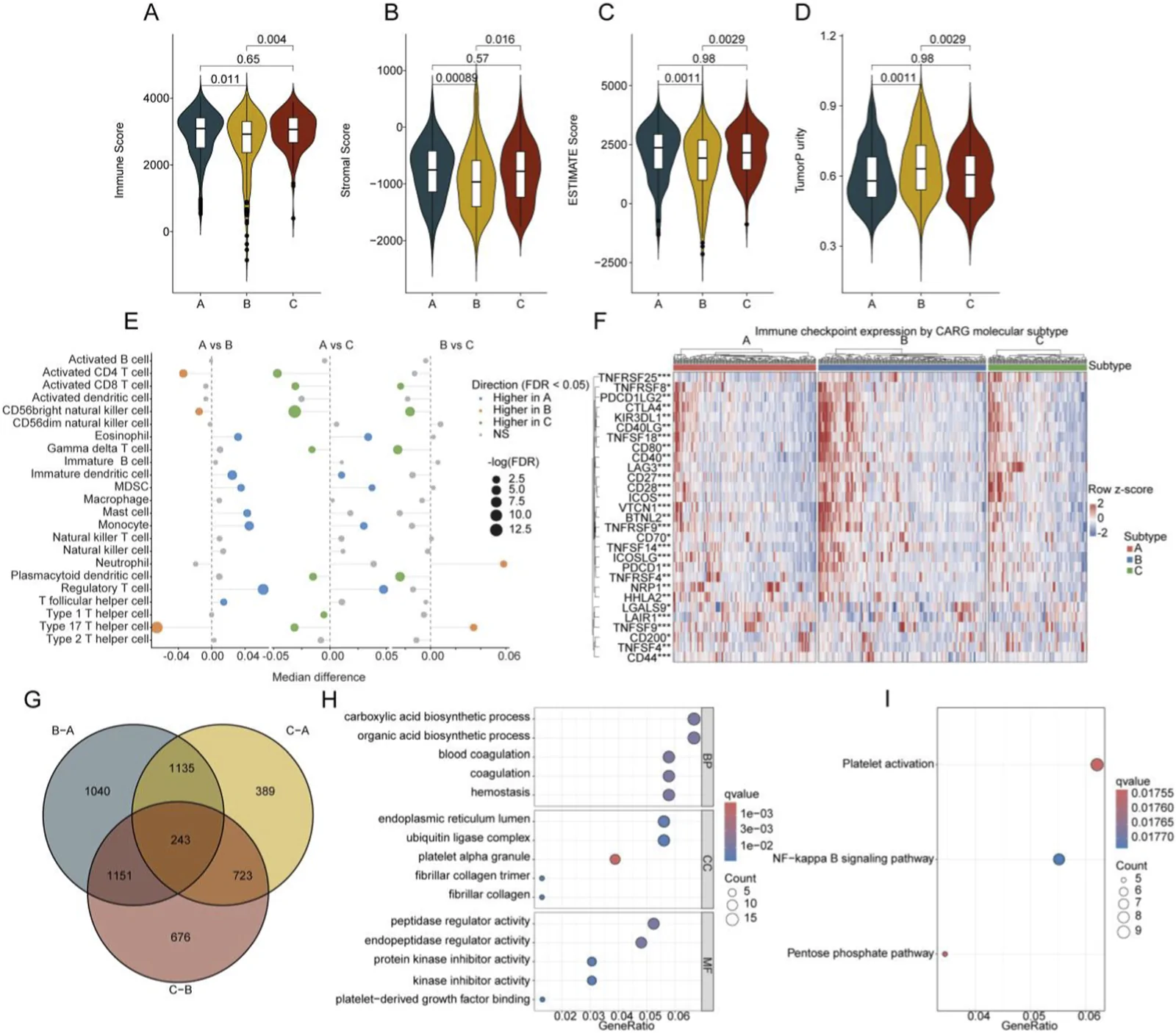
Immune microenvironment characteristics and functional enrichment of differentially expressed genes across CARG molecular subtypes. Analyses were performed in the integrated development cohort comprising TCGA-LAML (n = 130) and GSE37642 (n = 417; total n = 547). **(A–D)** Comparison of Immune Score, Stromal Score, ESTIMATE Score, and estimated tumor purity among the three CARG subtypes. **(E)** Pairwise comparisons of immune cell enrichment features across CARG subtypes. Dot color indicates the direction of enrichment, and dot size represents −log10(FDR). **(F)** Heatmap showing the expression patterns of immune regulatory genes across the CARG subtypes. **(G)** Venn diagram showing the overlap of differentially expressed genes identified from pairwise comparisons among the three subtypes, yielding 243 shared DEGs. **(H)** Gene Ontology (GO) enrichment analysis of the shared DEGs, including biological process (BP), cellular component (CC), and molecular function (MF) categories. **(I)** Kyoto Encyclopedia of Genes and Genomes (KEGG) pathway enrichment analysis of the shared DEGs.

### Integrated survival modeling establishes a CARG-based prognostic score and prioritizes SPINT2 in AML

The prognostic model was developed in the integrated development cohort comprising TCGA-LAML (n = 130) and GSE37642 (n = 417; total n = 547) and evaluated in the independent external validation cohort GSE12417 (n = 104). Patients with available overall survival time and survival status were included in the prognostic analyses (Supplementary Table S2). A total of 68 survival machine-learning strategies were evaluated and ranked according to their C-index performance in the development cohort (Supplementary Table S3). CoxBoost + SuperPC and Lasso + SuperPC showed the leading performance and were used to identify the final prognostic feature genes. External-validation and cross-cohort average C-indices were reported for descriptive comparison only and were not used for model selection (Figure 4A). SHAP analysis was subsequently used to quantify the contribution of individual genes to model predictions. Among the seven genes included in the final prognostic model, *SPINT2* exhibited the highest mean absolute SHAP value (0.125), followed by SH3BP5, SLC35C2, LAPTM4B, TMEM38B, AGTPBP1, and HIST1H4H (Figure 4B). Higher *SPINT2* expression was predominantly associated with positive SHAP values, indicating that increased *SPINT2* expression contributed to model predictions toward higher estimated risk. Based on the model-derived CARG prognostic score, patients were stratified into high- and low-score groups. High- and low-score groups were defined using the median CARG prognostic score within each respective cohort. In the development cohort, patients with high CARG prognostic score had significantly shorter OS than those with low scores (log-rank *P* < 0.001; Figure 4C). A consistent survival difference was observed in the external validation cohort, in which the high-score group also exhibited poorer OS (log-rank *P* = 0.035; Figure 4D). CARG prognostic score also differed significantly across the three CARG-defined molecular subtypes, with a progressive increase from Cluster A to Cluster C (Figure 4E). This gradient paralleled the corresponding survival differences among the three subtypes, demonstrating concordance between subtype-level stratification and the continuous patient-level prognostic score. Time-dependent receiver operating characteristic analysis further demonstrated AUCs of 0.751, 0.719, and 0.739 for 3-, 5-, and 7-year OS, respectively, in the development cohort (Figure 4F). In the external validation cohort, the corresponding AUCs were 0.644, 0.681, and 0.647 (Figure 4G). Although prognostic discrimination was lower in the external validation cohort than in the development cohort, survival remained significantly different between the cohort-specific median-defined score groups. Overall, the model showed significant survival separation in both cohorts, with more modest discrimination in external validation. SHAP analysis further identified *SPINT2* as the most influential feature contributing to model predictions, providing a rationale for its subsequent biological characterization.

**FIGURE 4:**
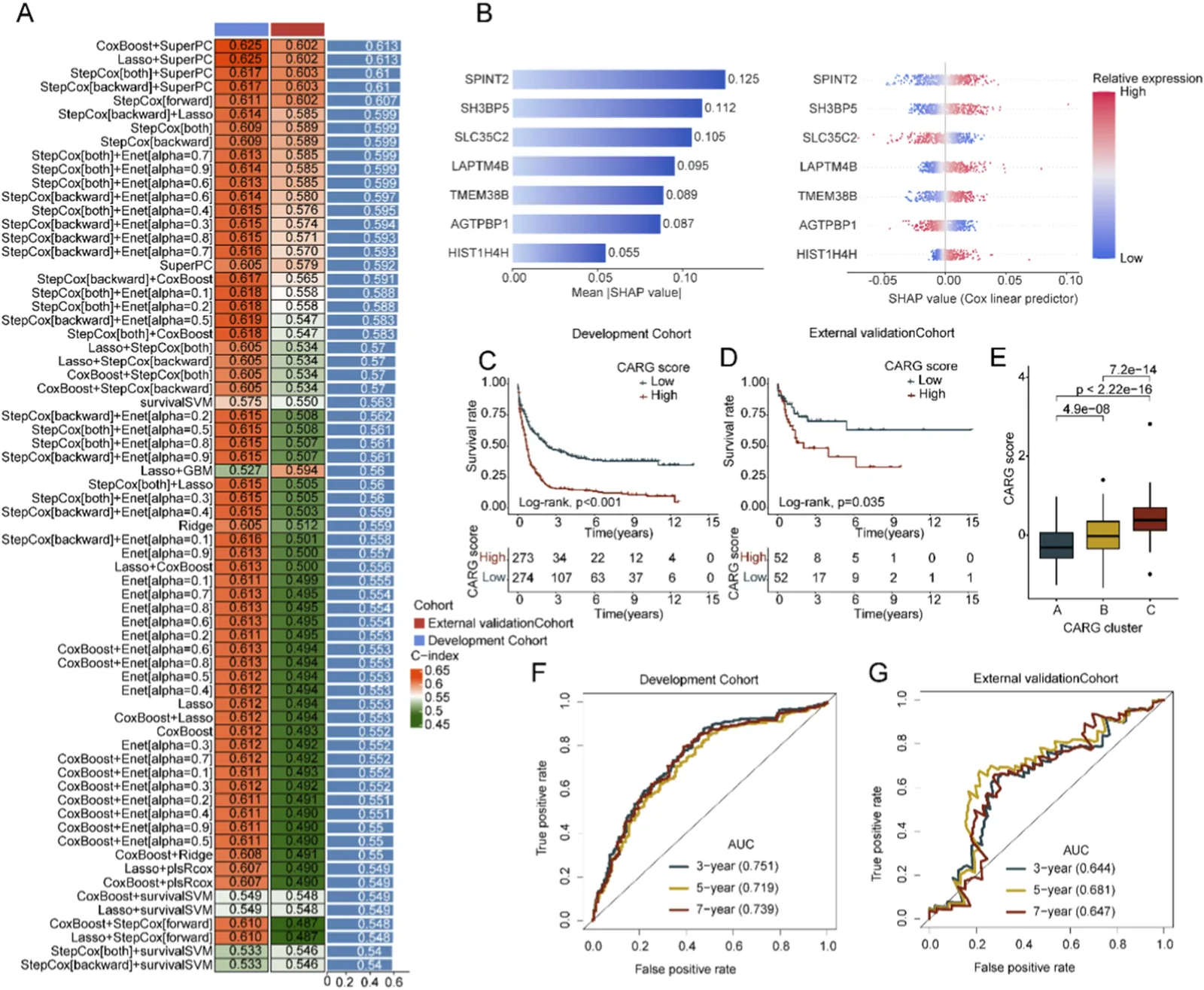
Multi-algorithm machine learning-based construction, interpretation, and validation of the CARG prognostic score. The model was developed in the integrated development cohort comprising TCGA-LAML (n = 130) and GSE37642 (n = 417; total n = 547) and evaluated in the independent external validation cohort GSE12417 (n = 104). **(A)** Comparison of C-indices for different machine-learning algorithms and combination models in the development cohort and independent external validation cohort. Models were ranked according to their C-index performance in the development cohort; cross-cohort average C-indices are shown for descriptive comparison only. **(B)** SHAP-based feature importance and summary plot for the seven genes included in the final prognostic model. **(C, D)** Kaplan–Meier curves comparing overall survival between the high- and low-CARG-score groups in the development and external validation cohorts, respectively, with groups defined using the cohort-specific median CARG prognostic score. **(E)** Distribution of CARG prognostic score across the three CARG molecular subtypes. **(F, G)** Time-dependent receiver operating characteristic (ROC) curves evaluating the performance of the CARG prognostic score for predicting 3-, 5-, and 7-year overall survival in the development and external validation cohorts, respectively.

### Clinical prognostic evaluation and survival prediction using the CARG prognostic score in AML

Clinical prognostic analyses were performed in the integrated development cohort comprising TCGA-LAML (n = 130) and GSE37642 (n = 417; total n = 547). Analyses involving FAB M3 status were restricted to patients with available FAB annotations. The CARG prognostic score was significantly associated with several clinical characteristics in patients with AML. Patients with non-M3 AML had higher CARG scores than those with FAB M3/M3v (APL-associated morphology) (*P* = 0.034), and CARG scores were also significantly higher in patients aged ≥65 years than in younger patients (Figures 5A,B). Univariable Cox regression analysis showed that FAB M3 status, age, and the CARG score were significantly associated with OS. In particular, increasing CARG prognostic score was associated with a higher risk of death (HR = 2.192, 95% CI: 1.823–2.636, *P* < 0.001; Figure 5C). In multivariable analysis adjusting for age and FAB M3 status, the CARG prognostic score remained significantly associated with OS (HR = 2.085, 95% CI: 1.716–2.533, *P* < 0.001). Age also remained statistically significant, whereas FAB M3 status did not retain significance after adjustment (*P* = 0.064; Figure 5D). These findings indicate that the CARG prognostic score remained associated with OS after adjustment for age and FAB M3 status within the available clinical variables. A nomogram integrating age and the CARG prognostic score was subsequently developed to estimate 3-, 5-, and 7-year OS probabilities (Figure 5E). Calibration curves showed close agreement between predicted and observed survival probabilities at all three time points. The corresponding Brier scores were 0.182, 0.172, and 0.160, with expected-to-observed (E/O) ratios of 0.996, 1.003, and 0.995 for 3-, 5-, and 7-year OS, respectively (Figures 5F–H). Time-dependent C-index analysis further showed that the CARG prognostic score generally achieved greater prognostic discrimination than age alone across follow-up (Figure 5I). Decision curve analysis demonstrated that the combined model incorporating age and the CARG prognostic score yielded greater net benefit than the age-only model across clinically relevant ranges of threshold probabilities for 3-, 5-, and 7-year survival prediction (Figures 5J–L). Collectively, these results indicate that the CARG score remained associated with OS after adjustment for age and FAB M3 status and may provide additional prognostic information beyond age alone within the clinical variables evaluated in this study.

**FIGURE 5:**
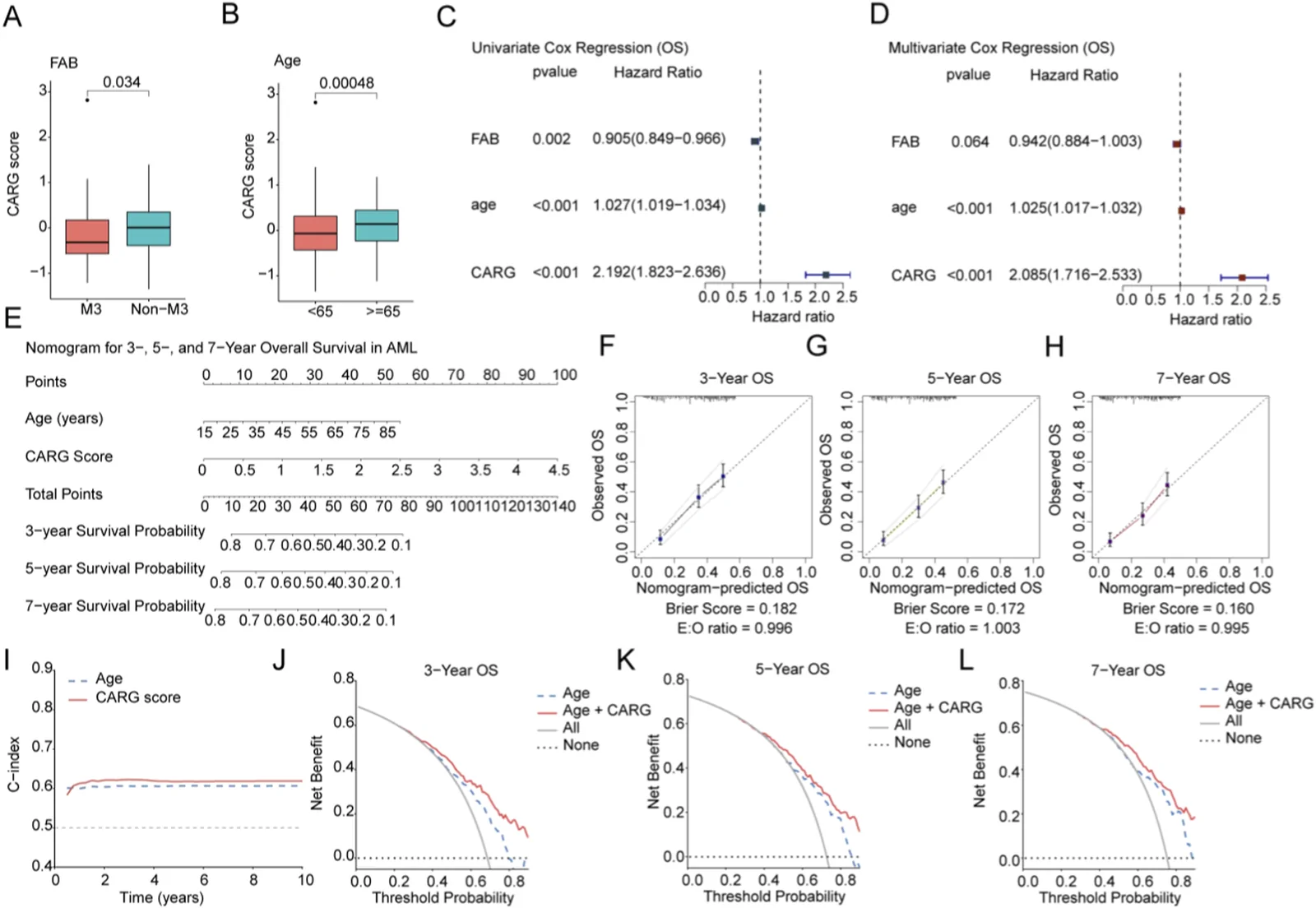
Clinical prognostic analyses were performed in the integrated development cohort comprising TCGA-LAML (n = 130) and GSE37642 (n = 417; total n = 547), with FAB-based analyses restricted to patients with available FAB annotations. **(A)** Distribution of CARG scores according to FAB M3 status [FAB M3/M3v, corresponding to acute promyelocytic leukemia (APL)-associated morphology, vs. non-M3 AML]. **(B)** Distribution of CARG scores according to age group. **(C, D)** Univariable and multivariable Cox regression analyses evaluating the associations of FAB M3 status, age, and the CARG score with overall survival. **(E)** Nomogram incorporating age and the CARG prognostic score for predicting 3-, 5-, and 7-year overall survival. **(F–H)** Calibration curves for the nomogram at 3, 5, and 7 years, with the corresponding Brier scores and expected-to-observed (E/O) ratios. **(I)** Comparison of the time-dependent concordance index (C-index) between age and the CARG prognostic score. **(J–L)** Decision curve analyses comparing the age-only model with the combined age plus CARG prognostic score model for predicting 3-, 5-, and 7-year overall survival.

### CARG risk stratification is associated with distinct immune-related profiles and ex vivo drug responses in AML

Immune-related analyses were first performed in the integrated development cohort comprising TCGA-LAML (n = 130) and GSE37642 (n = 417; total n = 547). CARG prognostic score-based risk stratification was associated with distinct immune-related transcriptional features. Compared with the low-CARG-score group, the high-score group showed relatively higher enrichment of signatures corresponding to activated CD4^+^ T cells, activated CD8^+^ T cells, CD56^bright^ natural killer cells, and plasmacytoid dendritic cells, whereas signatures associated with macrophages, myeloid-derived suppressor cells, regulatory T cells, and several innate immune cell populations were relatively reduced (Figure 6A). In parallel, multiple immune regulatory genes, including PDCD1, PDCD1LG2, LAG3, VTCN1, BTNL2, IDO1, and CD44, were differentially expressed between the high- and low-CARG-score groups (Figure 6B), further indicating distinct immune-related transcriptional states associated with CARG-defined risk. We next examined the relationship between the CARG prognostic score and *ex vivo* drug response using Beat AML 2.0 waves 1–4. The CARG prognostic score was significantly correlated with probit-curve area under the curve (AUC) values for multiple AML-relevant compounds (Figure 6C), with lower AUC values indicating greater *ex vivo* drug sensitivity. Higher CARG prognostic scores were positively correlated with AUC values for Venetoclax, GSK690693, Palbociclib, Sorafenib, and SB-431542, suggesting reduced *ex vivo* sensitivity to these agents. By contrast, Panobinostat and Selumetinib showed negative correlations between AUC and CARG prognostic score, indicating relatively greater sensitivity among samples with higher CARG prognostic score. Consistent with these correlation patterns, direct group comparisons showed that samples in the high-CARG-score group were less sensitive to Sorafenib, Venetoclax, GSK690693, and SB-431542, but more sensitive to Panobinostat and Selumetinib than those in the low-score group (Figure 6D). Exploratory class-level analysis showed that CARG-associated compounds were distributed across multiple pharmacological classes, including FLT3, MEK, AKT, EGFR/ERBB, VEGFR, RAF, BCL-2, CDK4/6, NF-κB, ALK, and MET inhibitors (Figure 6E). Although Panobinostat was individually associated with greater sensitivity in the high-CARG-score group, HDAC inhibitors were not displayed in the class-level analysis because Panobinostat was the only evaluable HDAC inhibitor meeting the analysis criteria. Collectively, these findings indicate that CARG-defined risk states are associated with distinct immune-related transcriptional profiles and differential *ex vivo* drug-response patterns, suggesting that the CARG prognostic score may capture biologically distinct AML states with potentially different pharmacological vulnerabilities.

**FIGURE 6:**
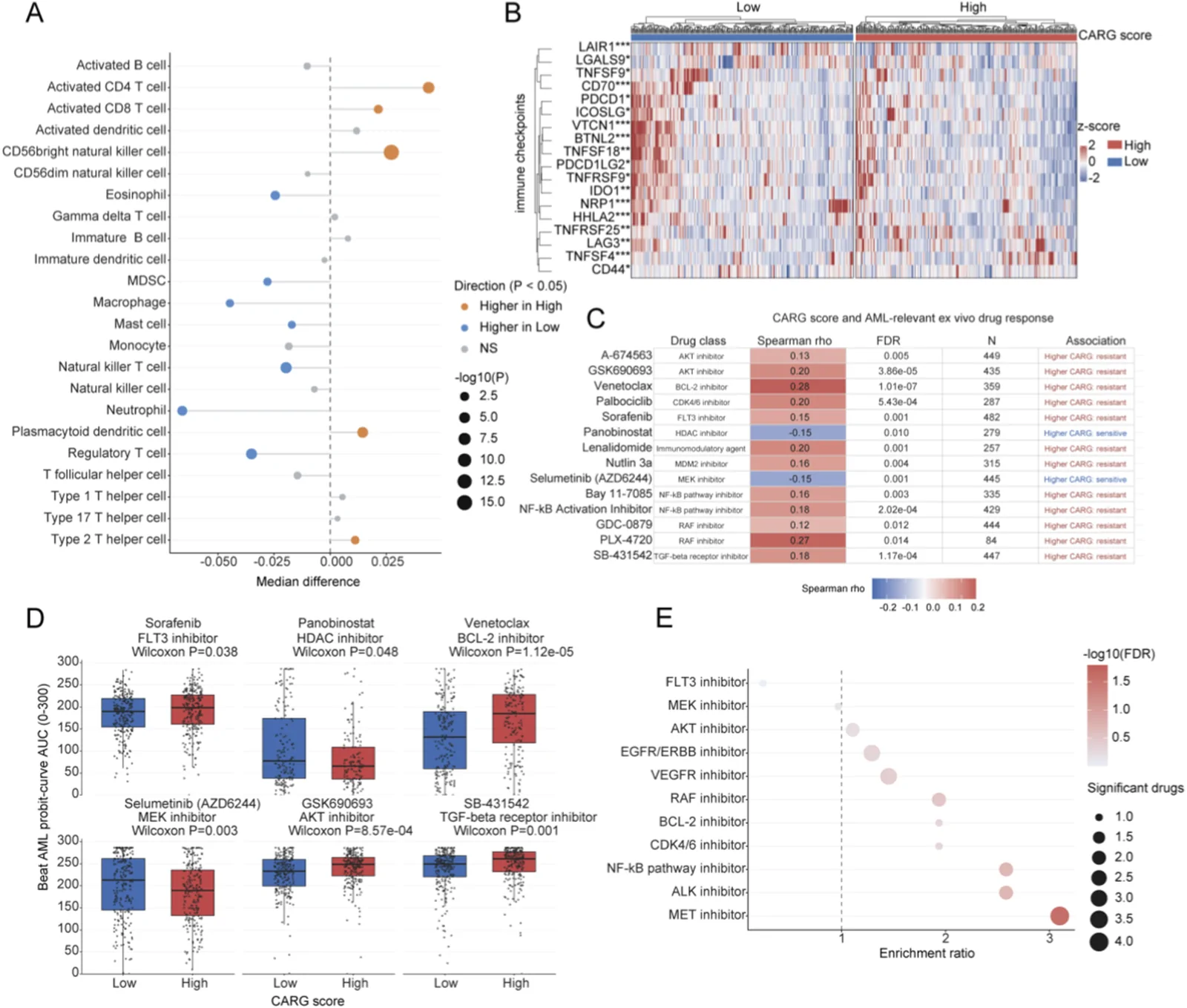
Immune analyses were performed in the integrated development cohort comprising TCGA-LAML (n = 130) and GSE37642 (n = 417; total n = 547), whereas *ex vivo* drug-response analyses were based on Beat AML 2.0 waves 1–4. **(A)** Comparison of immune cell enrichment features between the high- and low-CARG-score groups. Dot color indicates the direction of the difference, and dot size represents −log10(FDR). **(B)** Heatmap showing the expression patterns of immune regulatory genes in the high- and low-CARG-score groups. **(C)** Spearman correlation analysis between the CARG prognostic score and *ex vivo* responses to AML-related drugs. Lower Beat AML probit-curve area under the curve (AUC) value indicates greater drug sensitivity. **(D)** Comparison of probit-curve AUC values for representative drugs between the high- and low-CARG-score groups. **(E)** Exploratory class-level analysis of CARG-associated compounds. The x-axis represents the enrichment ratio, dot size indicates the number of CARG-associated drugs, and color represents −log10(FDR) from the one-sided Fisher’s exact test. Only drug classes containing at least two evaluable compounds and at least one CARG-associated drug are displayed.

### Single-cell profiling of *SPINT2* expression and the intercellular communication landscape in AML

Using the same single-cell dataset (GEO GSE289435; 12 primary AML samples), we next examined the cell type-specific distribution of the seven prognostic model genes. These genes exhibited heterogeneous expression patterns across the annotated cell populations. Among them, *SPINT2* showed relatively high expression in cycling erythroid cells, B cells, and dendritic cells (DCs) (Figure 7A). UMAP visualization and cell type-stratified analysis further confirmed the nonuniform distribution of *SPINT2*, with prominent expression in cycling erythroid cells and B cells and appreciable expression in DCs (Figures 7B,C). We next characterized intercellular communication among the major AML cell populations. Cell–cell communication analysis revealed an extensive and heterogeneous signaling network across the identified cellular compartments (Figures 7D–G). Monocytes, B cells, and T cells showed relatively strong outgoing and incoming signaling activity, whereas erythroid cells were characterized predominantly by higher incoming signaling strength (Figure 7H). Among the inferred signaling pathways, MIF exhibited the highest overall communication probability, followed by CyPA, CD99, and GALECTIN signaling (Figure 7I). Further analysis of the MIF network identified MIF–(CD74+CXCR4) and MIF–(CD74^+^CD44) as major ligand–receptor interactions contributing to communication across multiple cell populations (Figure 7J). Together, these analyses characterize the cell-type-dependent expression of *SPINT2* and independently delineate the broader intercellular communication landscape of AML, in which MIF signaling represented a prominent communication pathway.

**FIGURE 7:**
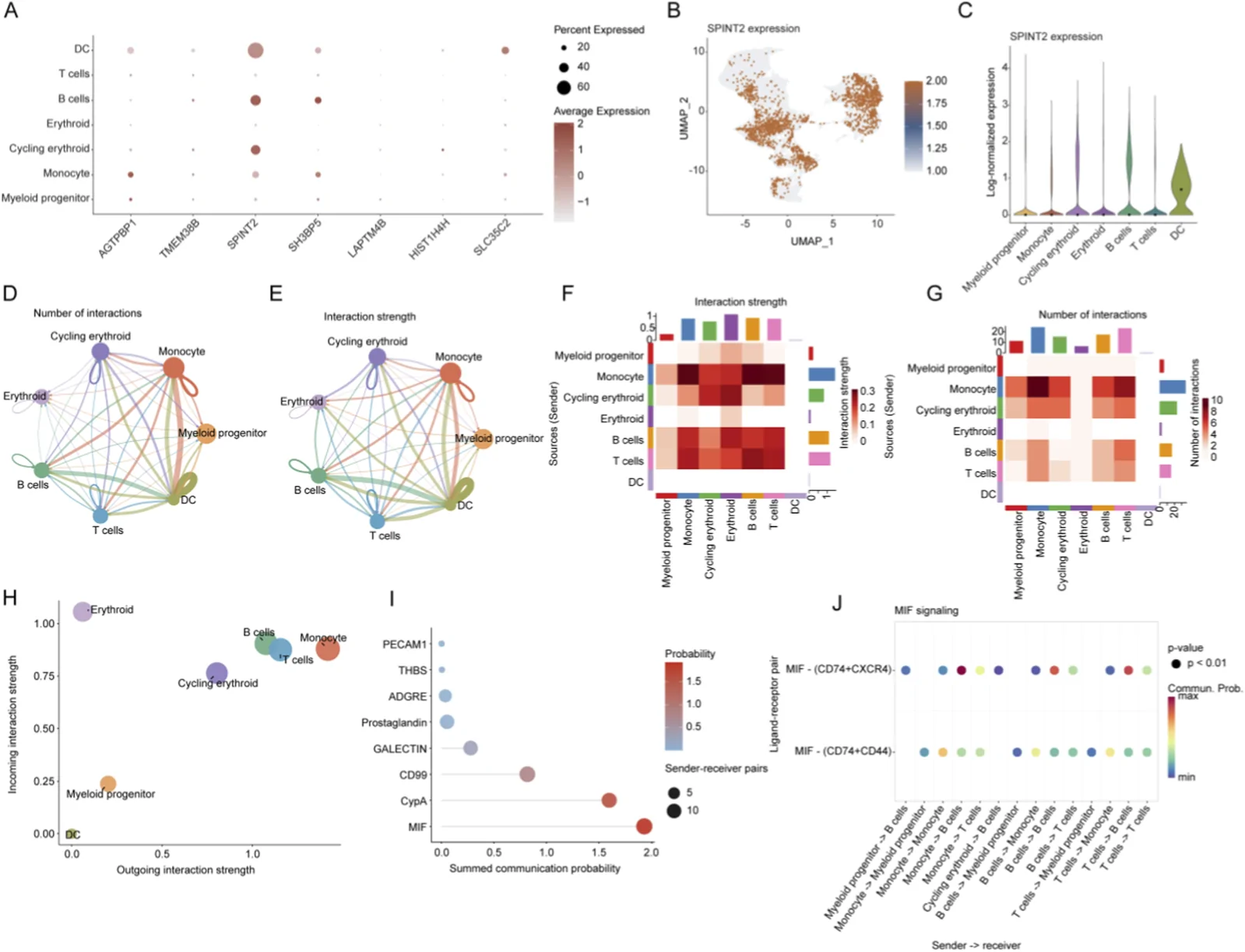
Single-cell expression characteristics of *SPINT2* and the intercellular communication network in AML. Analyses were based on GEO dataset GSE289435 comprising 12 primary AML samples. **(A)** Expression distributions of the seven prognostic model genes across the annotated cell populations. **(B)** UMAP feature plot showing the distribution of *SPINT2* expression at single-cell resolution. **(C)** *SPINT2* expression levels across the annotated cell populations. **(D, E)** CellChat analysis showing the number and overall strength of cell–cell interactions among the major cell populations. **(F, G)** Communication strength and interaction number of different cell populations acting as signal senders and receivers. **(H)** Distribution of outgoing and incoming signaling strengths across different cell types. **(I)** Overall communication probabilities and numbers of ligand–receptor pairs for the major signaling pathways. **(J)** Cell–cell communication patterns mediated by the MIF–(CD74+CXCR4) and MIF–(CD74^+^CD44) ligand–receptor axes within the MIF signaling network.

### Dual virtual-knockout analyses converge on erythroid, heme, and oxidative metabolic programs associated with SPINT2

Using the integrated whole-cell atlas derived from GEO GSE289435 (12 primary AML samples), we performed two independent computational virtual-knockout analyses of *SPINT2* using scTenifoldKnk and GenKI. scTenifoldKnk identified prominent network perturbation signals involving genes such as SLC4A1, GYPC, SLC25A37, BLVRB, PRDX2, and HMBS following *in silico SPINT2* knockout (Figure 8A). In parallel, GenKI prioritized MALAT1, EREG, AZU1, MTRNR2L12, and HSP90AA1 among the genes with relatively strong perturbation signals (Figure 8B). Although the two approaches differed in their gene-level rankings, increasing concordance was observed among broader sets of highly ranked candidates. Specifically, 5, 23, and 140 genes overlapped between the top 100, top 250, and top 500 candidates identified by scTenifoldKnk and GenKI, respectively (Figure 8C). Cross-method ranking analysis further identified SLC4A1, GYPC, BLVRB, SLC25A37, HBD, PRDX2, and TUBA1B among the genes consistently prioritized by both algorithms (Figure 8D). Functional enrichment analysis of the cross-method consensus genes revealed significant enrichment of Hallmark programs related to E2F and MYC targets, heme metabolism, fatty acid metabolism, and oxidative phosphorylation (Figure 8E). KEGG analysis similarly highlighted oxidative phosphorylation and pathways associated with mitochondrial function (Figure 8F), whereas Reactome analysis identified enrichment of neutrophil degranulation, the tricarboxylic acid cycle and respiratory electron transport, organelle biogenesis and maintenance, and HSF1-mediated stress-response programs (Figure 8G). Integration of ranking and perturbation evidence across both algorithms further highlighted TUBA1B, HBD, PRDX2, TUBB, GYPC, SLC25A37, SLC4A1, BLVRB, and HMBS as genes with strong cross-method support (Figure 8H). Collectively, scTenifoldKnk and GenKI showed only partial concordance at the individual-gene level but converged at the pathway level on erythroid/heme-associated processes, mitochondrial oxidative metabolism, and cellular stress-response programs. Because these analyses were performed across the integrated whole-cell atlas, these findings should be interpreted as computationally predicted transcriptional associations following *SPINT2* perturbation rather than evidence of lineage-specific or direct downstream mechanisms.

**FIGURE 8:**
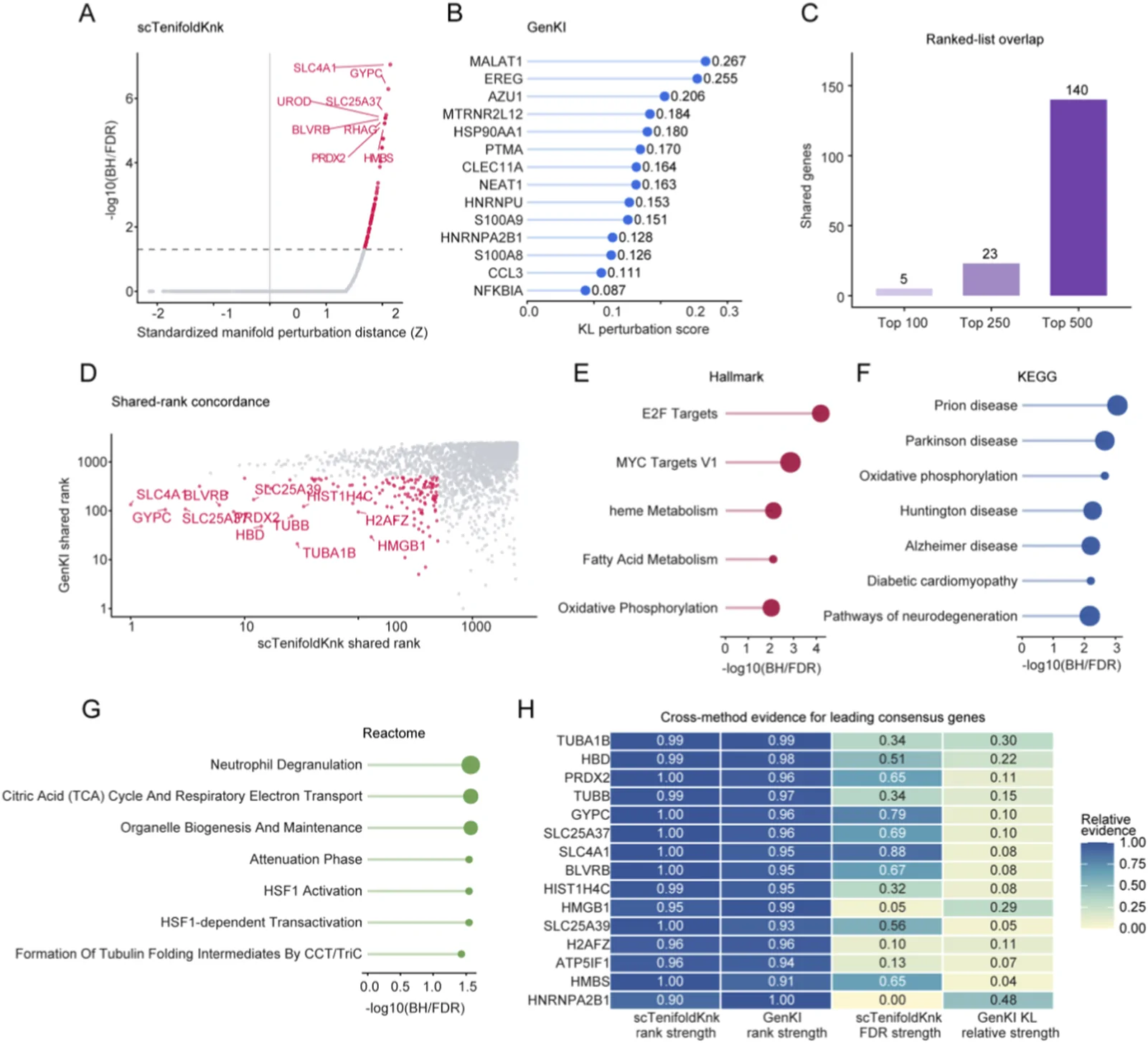
Dual-algorithm virtual knockout analysis reveals *SPINT2* perturbation-associated transcriptional networks and functional programs. Computational perturbation analyses were performed using the integrated single-cell atlas derived from GEO GSE289435 (12 primary AML samples). **(A)** Significantly perturbed genes identified by scTenifoldKnk following virtual knockout of *SPINT2*. **(B)** Top candidate genes ranked by KL perturbation score in the GenKI analysis. **(C)** Numbers of overlapping genes between the top 100, top 250, and top 500 candidates identified by scTenifoldKnk and GenKI. **(D)** Rank concordance of candidate genes shared between the two virtual knockout approaches. **(E–G)** Hallmark, KEGG, and Reactome pathway enrichment analyses of cross-method consensus candidate genes. **(H)** Integrated cross-method evidence for major consensus candidate genes, including ranking strength, scTenifoldKnk FDR, and GenKI KL perturbation scores.

### SPINT2 silencing suppresses AML cell proliferation and promotes apoptosis

We next evaluated the phenotypic consequences of *SPINT2* depletion in AML cells. *SPINT2* mRNA expression was first examined across a panel of AML cell lines. Relative to 293T cells, *SPINT2* expression was significantly higher in HL-60, Kasumi-1, KG-1, MOLM-13, OCI-AML3, and THP-1 cells, with the highest expression observed in MOLM-13 cells (Figure 9A). Western blot analysis further confirmed detectable *SPINT2* protein expression across these AML cell lines (Figure 9B). Based on their endogenous *SPINT2* expression profiles, THP-1 and MOLM-13 cells were selected to evaluate the phenotypic effects of *SPINT2* silencing. *SPINT2* was silenced using two independent shRNAs, and Western blotting confirmed efficient reduction of *SPINT2* protein levels by both sh*SPINT2*#1 and sh*SPINT2*#2 in THP-1 and MOLM-13 cells (Figure 9C). Two-way ANOVA revealed significant effects of treatment, time, and treatment × time interaction on the proliferation profiles of both THP-1 and MOLM-13 cells. Compared with shNC controls, *SPINT2*-knockdown cells showed progressively lower relative absorbance values over the culture period, with significant differences becoming evident at later time points (Figures 9D,E). Consistently, EdU incorporation assays demonstrated a marked reduction in the proportion of EdU-positive cells following *SPINT2* silencing in both THP-1 and MOLM-13 cells (Figures 9F–H), indicating impaired DNA synthesis and proliferative activity. Annexin V/PI flow cytometric analysis further showed that *SPINT2* knockdown significantly increased the proportion of apoptotic cells in both AML cell lines (Figures 9I–K). Collectively, these findings demonstrate that *SPINT2* silencing inhibits AML cell proliferation and enhances apoptosis, supporting a role for *SPINT2* in maintaining AML cell growth and survival.

**FIGURE 9:**
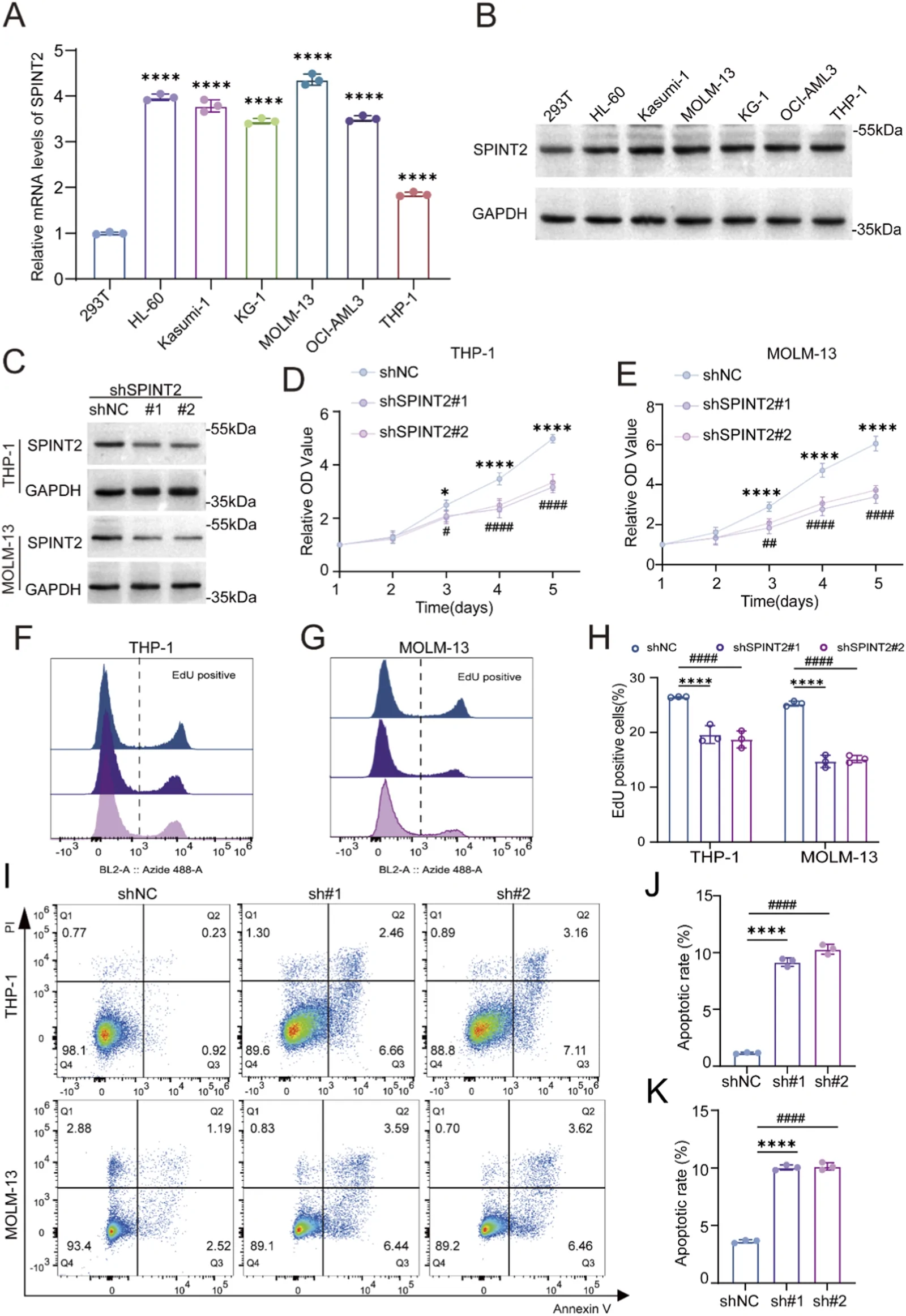
*SPINT2* silencing suppresses AML cell proliferation and promotes apoptosis. **(A)** RT-qPCR analysis of *SPINT2* mRNA expression in 293T cells and different AML cell lines. **(B)** Western blot analysis of *SPINT2* protein expression across the indicated cell lines, with GAPDH used as the loading control. **(C)** Western blot validation of *SPINT2* knockdown efficiency by sh*SPINT2*#1 and sh*SPINT2*#2 in THP-1 and MOLM-13 cells. **(D, E)** Time-course analysis of cell proliferation in THP-1 and MOLM-13 cells following *SPINT2* knockdown. CCK-8 data were analyzed separately for each cell line using ordinary two-way ANOVA with treatment and time as independent factors, including the treatment × time interaction, followed by Dunnett’s multiple-comparison test comparing sh*SPINT2*#1 and sh*SPINT2*#2 with shNC at individual time points. **(F, G)** EdU incorporation assays evaluating proliferative activity in THP-1 and MOLM-13 cells after *SPINT2* knockdown. **(H)** Quantification of the proportion of EdU-positive cells. **(I)** Annexin V/PI flow cytometric analysis of apoptosis in THP-1 and MOLM-13 cells following *SPINT2* knockdown. **(J, K)** Quantification of the apoptotic fraction in THP-1 and MOLM-13 cells. Data are presented as the mean ± SD from three independent experiments (n = 3). For panel A, ^****^ *P* < 0.0001 versus 293T. For panels D–K, ^*^ *P* < 0.05, ^**^ *P* < 0.01, ^***^ *P* < 0.001, and ^****^ *P* < 0.0001 indicate sh*SPINT2*#1 versus shNC; ^#^ *P* < 0.05, ^##^ *P* < 0.01, ^###^ *P* < 0.001, and ^####^ *P* < 0.0001 indicate sh*SPINT2*#2 versus shNC.

## Discussion

In the present study, we systematically investigated the role of CARGs in AML and demonstrated that they define distinct molecular subtypes with significant differences in prognosis, metabolic activity, and immune microenvironment characteristics. By integrating multi-cohort transcriptomic data, we established a CARG-based prognostic scoring system that showed consistent but modest prognostic discrimination across cohorts and remained associated with OS after adjustment for age and FAB M3 status. Notably, our findings further revealed a close association between coagulation-related signatures and immune dysregulation, as well as identified *SPINT2* as a candidate functional determinant whose role in AML cell growth and survival was supported by *in vitro* experiments. These results provide a comprehensive framework linking coagulation pathways to AML progression and offer new insights into risk stratification and potential therapeutic intervention strategies.

At the single-cell level, our analysis revealed substantial heterogeneity in CARG-related transcriptional activity across AML cellular populations, with the highest relative CARG UCell activity observed in cycling erythroid and erythroid cells, whereas myeloid progenitors, monocytes, and immune populations showed more heterogeneous patterns. This finding is consistent with the recognized cellular complexity of AML, in which malignant and non-malignant populations exhibit distinct transcriptional states and differentiation programs (Saadatpour et al., 2014; van Galen et al., 2019). Previous single-cell studies have further demonstrated substantial interpatient variation in cellular composition and transcriptional states, which can be associated with genetic features, immune phenotypes, treatment response, and clinical outcomes (Zeng et al., 2022; Zhang et al., 2024). In this context, the preferential enrichment of CARG-related transcriptional activity in erythroid and cycling erythroid populations suggests that the CARG signal may be associated with specific hematopoietic cell states rather than being uniformly distributed across AML cells. Importantly, the CARG UCell activity score reflects the relative transcriptional activity of a predefined gene set and does not directly measure coagulation activity or procoagulant function. This distinction is particularly relevant in AML, where coagulation abnormalities arise from complex interactions involving leukemic cells, blood cells, tissue factor-related pathways, microparticles, and the hematopoietic environment (Langer et al., 2004; Dicke et al., 2015). Thus, the higher CARG UCell activity in erythroid populations should be interpreted as enrichment of coagulation-associated transcriptional programs rather than evidence of direct coagulation activation. Because bulk transcriptomic profiles integrate signals from multiple cellular populations, variation in cellular composition may also contribute to differences in bulk CARG signatures between patients (Dai et al., 2021; Wang et al., 2022; Lilljebjorn et al., 2025). Previous work has shown that cellular hierarchy inferred from bulk AML transcriptomes can capture clinically relevant biological variation, including treatment response and drug sensitivity (Zeng et al., 2022). AML-associated remodeling of the bone marrow microenvironment further adds cellular and intercellular complexity to these transcriptional profiles (Guo et al., 2021; Ennis et al., 2023). Taken together, our findings provide a cellular context for bulk CARG signatures and suggest that CARG-associated heterogeneity may partly reflect differences in cellular composition and cell-state-specific transcriptional programs across AML patients.

The enrichment of carboxylic acid biosynthetic process and organic acid biosynthetic process in association with AML risk stratification suggests that metabolic reprogramming plays a pivotal role in disease heterogeneity and clinical outcomes (Addanki et al., 2025; Hao et al., 2025; Raimondi et al., 2022). Carboxylic and organic acids serve as key intermediates in fundamental metabolic pathways, including the tricarboxylic acid (TCA) cycle, amino acid metabolism, and lipid biosynthesis, which are essential for supporting the bioenergetic and anabolic demands of leukemic cell proliferation (Kreitz et al., 2019; Xiao et al., 2023; Yu and Yan, 2025). Emerging evidence indicates that metabolic dependencies differ across AML subtypes and risk groups, with high-risk patients often exhibiting increased reliance on mitochondrial metabolism and oxidative phosphorylation, potentially contributing to therapy resistance (Pereira-Martins et al., 2025; Farge et al., 2017). Notably, recurrent mutations such as IDH1 and IDH2 directly disrupt organic acid metabolism through the production of the oncometabolite 2-hydroxyglutarate, leading to epigenetic dysregulation and impaired differentiation (Figueroa et al., 2010; Garrett-Bakelman and Melnick, 2016). In addition, alterations in these biosynthetic processes may influence redox balance and reactive oxygen species homeostasis, thereby affecting leukemic stem cell maintenance and drug sensitivity (Griessinger et al., 2023; Farge et al., 2017). Collectively, these findings may suggest that the enrichment of carboxylic and organic acid biosynthetic pathways may reflect underlying metabolic adaptations linked to AML risk stratification and highlight their potential value in refining prognostic models (Peng et al., 2022).

Building on these metabolic insights, CARGs may further contribute to the heterogeneity of treatment response and drug resistance in AML. Increasing evidence indicates that components of the coagulation cascade are not merely passive bystanders but actively participate in leukemic progression through crosstalk with inflammatory signaling, endothelial activation, and the bone marrow microenvironment (Fodil et al., 2022; Bakhtiyari et al., 2023). For instance, tissue factor (TF) and other coagulation mediators can activate protease-activated receptors (PARs), thereby triggering downstream pathways such as MAPK and PI3K/AKT that promote leukemic cell survival and resistance to apoptosis (Aberg and Siegbahn, 2013; Goyama et al., 2017). In addition, coagulation-related factors can enhance the formation of a protective niche by modulating endothelial cells and stromal interactions, which in turn reduces chemotherapy sensitivity and facilitates minimal residual disease persistence (Falanga et al., 2013; Titah et al., 2026). Hypercoagulability is also associated with increased cytokine release and chronic inflammation, conditions that may reinforce leukemic stem cell maintenance and contribute to immune evasion (Wang et al., 2025). Furthermore, the contribution of coagulation proteases to cancer immune evasion provides a mechanistic basis for the link between coagulation-related molecular mechanisms and immune evasion (Paul et al., 2024). Therefore, this evidence, in line with our results, shows that CARGs may represent an additional layer of biological complexity underlying AML risk stratification and provide novel insights into mechanisms of therapeutic resistance.

Our integrative analysis revealed that the high-CARG-score group exhibited higher enrichment scores for transcriptional signatures corresponding to activated CD4^+^ T cells, activated CD8^+^ T cells, and plasmacytoid dendritic cells (pDCs). These ssGSEA-derived differences reflect relative enrichment of immune cell-associated transcriptional programs rather than direct measurements of cellular abundance or functional state. Nevertheless, previous studies have shown that CD8^+^ T cells in AML may display an exhausted phenotype characterized by impaired effector function and reduced cytotoxic capacity (Knaus et al., 2018; Wang et al., 2024). Such dysfunctional CD8^+^ T cells are unable to efficiently eliminate leukemic blasts despite their increased abundance, thereby contributing to disease persistence and progression (Tasis et al., 2025). Moreover, the leukemic bone marrow microenvironment further promotes T-cell dysfunction through immunosuppressive signaling and impaired antigen presentation, facilitating immune escape and adverse clinical outcomes (Tan et al., 2020). In parallel, the increased infiltration of pDCs in the high-risk group may further contribute to an immunosuppressive microenvironment. Previous studies have demonstrated that pDCs can infiltrate AML bone marrow and define biologically distinct disease subsets (Xiao et al., 2021). Importantly, pDCs in AML often exhibit a tolerogenic or functionally impaired phenotype, including reduced type I interferon production and weakened immune activation capacity (Fatehchand et al., 2021). Functionally, such dysfunctional pDCs may fail to effectively prime anti-leukemic T-cell responses and instead contribute to immune tolerance within the leukemia microenvironment. In addition, accumulation of pDCs has been associated with more aggressive disease features and unfavorable clinical outcomes in AML (Wang et al., 2017; El Hussein and Wang, 2023). Collectively, the coexistence of exhausted CD8^+^ T cells and immunoregulatory pDCs may provide a biological context for the observed association between increased immune infiltration and poor prognosis observed in our cohort, emphasizing that immune cell functional states, rather than mere abundance, are critical determinants of AML progression and risk stratification.

At single-cell resolution, *SPINT2* exhibited a heterogeneous, cell-type-dependent expression pattern, with relatively high expression in cycling erythroid cells, B cells, and DCs. This distribution should be distinguished from the CARG UCell activity profile because the two measurements represent different analytical levels: the former reflects expression of an individual prognostic gene, whereas the latter summarizes the activity of a predefined gene set. Notably, cycling erythroid cells exhibited relatively high levels of both *SPINT2* expression and CARG activity, indicating partial concordance rather than a direct one-to-one correspondence. This heterogeneous distribution suggests that the biological relevance of *SPINT2* in AML may be associated with specific cellular states rather than uniformly shared across all cell populations. Such cell-state specificity may also contribute to its prognostic association in bulk transcriptomic cohorts, which integrate expression signals from multiple cellular compartments. *SPINT2* (serine peptidase inhibitor, Kunitz type 2), also known as hepatocyte growth factor activator inhibitor-2 (HAI-2), is a membrane-associated serine protease inhibitor that primarily regulates pericellular proteolysis, including the activation of hepatocyte growth factor (HGF) and downstream MET signaling (Roversi et al., 2018). Dysregulation of the HGF/MET axis has been implicated in leukemogenesis, promoting proliferation, survival, and resistance to apoptosis in AML (Boissinot et al., 2014; Kentsis et al., 2012). In this context, our findings that high *SPINT2* expression is associated with inferior prognosis suggest a potential pro-tumorigenic role in AML, which appears paradoxical given its classical characterization as a tumor suppressor in several solid malignancies, such as gastric cancer, renal cell carcinoma, and medulloblastoma (Dong et al., 2010; Morris et al., 2005; Kongkham et al., 2008). This discrepancy may reflect the context-dependent functions of *SPINT2* within distinct cellular and microenvironmental settings (Roversi et al., 2018). Functionally, our *in vitro* data demonstrate that *SPINT2* knockdown significantly suppresses AML cell proliferation while promoting apoptosis, indicating that *SPINT2* may contribute to leukemic cell survival. The computational perturbation results should be distinguished from the phenotypic validation in THP-1 and MOLM-13 cells. The virtual-knockout analyses were performed across the integrated single-cell atlas and identified transcriptional programs associated with predicted *SPINT2* perturbation, including erythroid/heme-related and oxidative metabolic processes. By contrast, the *in vitro* experiments evaluated the effects of *SPINT2* depletion on AML cell proliferation and apoptosis. Therefore, the current data do not establish that these phenotypic effects are mediated by the specific genes or metabolic pathways highlighted by the computational analysis, which will require further lineage-resolved and mechanistic validation. Importantly, the MIF signaling network identified by CellChat was analyzed independently of the *SPINT2* perturbation analysis and should not be interpreted as a downstream *SPINT2*-mediated pathway. THP-1 and MOLM-13 cells were selected on the basis of their endogenous *SPINT2* expression to evaluate the general phenotypic consequences of *SPINT2* depletion in AML cells rather than to validate an erythroid-lineage-specific mechanism. Accordingly, erythroid-lineage models such as HEL or K562 were not included in the present study, and lineage-specific validation will be required to determine whether the computationally predicted erythroid/heme-related programs are functionally dependent on *SPINT2*. Beyond the computationally predicted pathways identified here, previous evidence suggests that *SPINT2* may also influence protease-dependent signaling cascades relevant to AML cell growth, potentially through modulation of HGF activation or other serine protease networks involved in extracellular matrix remodeling and cytokine activation (Roversi et al., 2019). Taken together, our findings support a context-dependent functional association between *SPINT2* and AML cell proliferation and survival. Although *SPINT2* may represent a candidate biomarker with potential biological relevance, further mechanistic and *in vivo* studies are required before its therapeutic relevance can be established. In conclusion, our study provides an integrative characterization of CARG-associated heterogeneity in AML, linking CARG-related transcriptional patterns with molecular subtypes, prognosis, metabolic programs, and immune-related features. The CARG-based scoring system showed consistent but modest prognostic discrimination across cohorts, while *SPINT2* emerged as a candidate gene with potential functional relevance in AML. Nevertheless, several limitations should be acknowledged. First, although multiple public datasets were integrated, the retrospective nature of the study may introduce inherent selection bias. Second, complete cytogenetic and molecular information required for ELN 2022 risk classification was not consistently available across the integrated cohorts, precluding a reliable direct comparison between the CARG prognostic score and ELN 2022 risk stratification. Therefore, whether the CARG prognostic score provides incremental prognostic value beyond established AML genetic risk classification remains to be determined in independent cohorts with comprehensive molecular and cytogenetic annotation. Prospective validation in larger populations is also warranted. Third, the mechanistic role of key genes, particularly *SPINT2*, was only partially explored, and further investigations of downstream pathways and *in vivo* functions are required. Finally, the complexity of the bone marrow microenvironment and its dynamic interactions with coagulation-associated processes remain incompletely understood.

## Data Availability

The original contributions presented in the study are included in the article/Supplementary Material, further inquiries can be directed to the corresponding author.
